# Spatial distribution and geospatial modeling of potential spread of secondary malaria vectors species in Nigeria using recently collected empirical data

**DOI:** 10.1371/journal.pone.0320531

**Published:** 2025-04-21

**Authors:** Adedapo O. Adeogun, Ayodele Samuel Babalola, Okoko Okefu Oyale, Tolulope Oyeniyi, Ahmed Omotayo, Romoke Tawakalitu Izekor, Oluwakemi Adetunji, Abiodun Olakiigbe, Olalekan Olagundoye, Monsuru Adebayo Adeleke, Chioma Cynthia Ojianwuna, Adamu Dagona, Daskum Abdullahi Muhammad, Jibrin Musa Mabu, Elkanah Obadiah Sambo, Adedayo Oduola, Petrus Uchenna Inyama, Lazarus Samdi, Abiodun Obembe, Mustapha Musa Dogara, Kennedy Poloma Yoriyo, Suleiman Mohammed, Rebecca Naphtali Samuel, Chioma Amajoh, Adesola Musa, Musa John Zabiri, Njobdi Sani, Sani Zakariya, Abubakar Samaila, Ezra Abba, Abdulmalik Bala Shuaibu, Victor Enwemiwe, Eric Esiwo, Ahmad Danjuma, Tasiu Shuaibu, Peni Aiki Istifanus, Salisu Kabiru, Azubuike Christian Ukubuiwe, Ibrahim Maikudi Salihu, Julius Akolawole Bamidele, Jumoke Kikelomo Fawole, Garba Columbus Liatu, Alex Jasini Wahedi, Sambo Fatima Idris, Abduljalal Ado, Micah Sale Pukuma, Kanil Ayo Fasasi, Akinlabi Muhammed Rufai, Ifeoluwa Kayode Fagbohun, Mohammed Bala, Mary Esema, Mamudu Omo-Eboh, Olufunmilayo Ajoke Idowu, Adeolu Ande, Israel Kayode Olayemi, Abdulsalami Manu Yayo, Cyril Ademu, Chukwu Okoronko, Lynda Ozor, James Ssekitooleko, Olugbenga Mokuolu, Issa Kawu, Godwin Ntadom, Babatunde Salako, Samson Awolola

**Affiliations:** 1 Nigerian Institute of Medical Research, Yaba, Lagos, Nigeria; 2 National Malaria Elimination Program, Federal Ministry of Health, Abuja, Nigeria; 3 Department of Zoology, Faculty of Basic and Applied Sciences, Osun State University, Osogbo, Nigeria; 4 Department of Animal and Environmental Biology, Delta State University, Abraka, Delta State, Nigeria; 5 Biology Research Laboraroty, Federal University, Gashua, Yobe State, Nigeria; 6 Department of Biological Sciences, Yobe State University, Damaturu, Nigeria; 7 Department of Biological Sciences, Taraba State University, Jalingo, Nigeria; 8 ABT Associates, Keffi, Nasarawa State, Nigeria; 9 Department of Zoology, Kwara State University, Malete, Kwara, Nigeria; 10 Department of Biological Sciences, Faculty of Science, Federal University, Jigawa, Dutse, Nigeria; 11 Department of Zoology, Faculty of Science, Gombe State University, Gombe, Nigeria; 12 Department of Biology, Umaru Musa Yar’adua University, Batagarawa, Katsina State, Nigeria; 13 Department of Zoology, Madibbo Adama University of Technology, Yola, Adamawa State, Nigeria; 14 Community Vision, Abuja, Nigeria; 15 Department of Biological Sciences, College of Education Hong, Jimeta, Adamawa State, Nigeria; 16 Department of Biological Sciences, Federal University, Dutsinma, Katsina State, Nigeria; 17 Department of Animal Biology, Federal University of Technology, Minna, Nigeria; 18 Department of Pure and Applied Zoology, Federal University of Agriculture, Abeokuta, Nigeria; 19 Centre for Infectious Diseases Research, Bayero University, Kano, Nigeria; 20 Arctech Innovation Limited, The Cube, Londoneast-uk Business and Technical Park, Dagenham, United Kingdom; 21 Department of Zoology, University of Ilorin, Ilorin, Kwara State, Nigeria; 22 World Health Organization, Abuja, Lagos State, Nigeria; 23 Global Fund, Abuja, Federal Capital Territory, Nigeria; 24 Faculty of Health Sciences, College of Health Sciences, University of Ilorin, Ilorin, Kwara State, Nigeria; Clinton Health Access Initiative, UNITED STATES OF AMERICA

## Abstract

In Nigeria, most research and malaria vector control efforts have focused on primary vectors within the *Anopheles gambiae* complex, with less emphasis on other secondary vectors. Consequently, understudied secondary vectors have demonstrated a proportional and increasing role in transmission. This study utilized geospatial models to understand the potential distribution of anopheline species other than *An*. *gambiae* complex (non-*gambiae* species) in Nigeria. Adult mosquitoes were sampled monthly between 2020 and 2022, with concurrent surveys of larval sites in selected Local Government Areas (LGAs) across 20 States resulting in the collection and identification of over 100,000 Anopheline mosquitoes. Utilizing 23 environmental variables, the model produced maps depicting the potential geographical distribution of four secondary vector species under current climatic conditions. *An*. *funestus*, *An*. *coustani*, *An*. *maculipalpis*, and *An*. *rufipes* dominated collections, with other species also present. Most species collected exhibited higher occurrences in the Northern parts of the country, albeit with lower numbers, while they seem confined to fewer locations in the southern parts - with higher densities. *An*. *funestus*, *An*. *maculipalpis*, and *An*. *rufipes* demonstrated a higher potential for wide range expansion compared to *An*. *coustani* based on the model. Overall, modeling outputs indicate that non-*An*. *gambiae* were expected to exhibit a wide-spread across the country, with their distribution primarily influenced by temperature rather than precipitation-related factors. These models provide research scientists and decision-makers with a baseline for research, monitoring towards establishing management plans for future national mosquito surveillance and control programs in Nigeria.

## Introduction

Malaria continues to be the most important tropical infectious diseases, with high prevalence Globally. In the year 2022, malaria resulted in more than 249 million reported cases in 85 endemic countries with Pakistan, Ethiopia, Nigeria, Uganda and Paupau New Guinea contributing heavily to these numbers [1]. In the WHO Africa Region, which accounted for about 94% of cases globally, case incidence increased from 226 per 1000 population at risk in 2019 to 232 per 1000 in 2020 [1] despite Cabo Verde reporting zero indigenous cases for 4 consecutive years. Nigeria account for 27 and 31% of global cases and deaths, respectively [1], making malaria a significant public health burden in Nigeria. The more recent localized National Malaria Indicator survey (MIS) also tells the tale^2^. MIS showed a steady but marginal decrease in number of children positive for malaria between 2015 (27%) and 2021 (22%), with increased efforts in ITN ownership, Intermittent Preventive Treatment during Pregnancy and Case management [2].

Malaria transmission across Africa exhibits significant heterogeneity owing to eco-climatic variations across the continent [3]. Currently, malaria infection in humans is attributed to five species of the *Plasmodium* parasites. Primary vector species, such as *Anopheles gambiae* sensu lato (s.l.) [4], *Anopheles funestus* group, and *Anopheles nili* group [5–7], primarily transmit the *Plasmodium* species causing human malaria in Africa. In Nigeria, as in most other African countries, malaria is primarily caused by *Plasmodium falciparum*, although other species are present at lower prevalence levels [1].

It has been proven that the level of malaria endemicity in any given region is influenced by the species of native anopheline mosquitoes and their relative abundance, among other factors [8]. Anopheline bionomics and diversity may have serious epidemiological implications to malaria transmission as it opens the avenue for emergence of new and more efficient vectors which could further complicate malaria transmission intensity [9]. Such complications could lead to extended malaria transmission seasons and expanded geographical distribution, as some invasive *Anopheles* species are also influencing malaria endemicity in certain regions—for instance, the recent spread of *Anopheles stephensi* in the African region [9,10].

To achieve malaria elimination in Nigeria, there is a need to extend surveillance strategies to other secondary malaria vectors that have not received significant attention until now. Even if the major malaria vectors (*An. gambiae* s.l.) in Nigeria, most of whose members bite indoors, are controlled at certain times, intermittent surges in the emergence of other Anopheline species could alter the malaria transmission landscape, complicating malaria control efforts. Moreover, to date, the core interventions of insecticide-treated nets (ITNs) and indoor residual spraying (IRS) are primarily deployed to target indoor malaria vectors. As a result, understanding the distribution of these other anopheline vectors, as well as the ecological factors responsible for their presence, is a valuable tool for combating malaria in the country.

In Nigeria, *Anopheles gambiae* s.l. has been reported as a major malaria vector with the widest distribution and abundance [10–12]. However, other vectors including *Anopheles coustani, Anopheles funestus*, and *Anopheles moucheti* have also been reported as prevalent species responsible for transmitting malaria parasites in pockets [13–15] across different regions of the country.

The absence of contemporary *Anopheles* distributional data has consistently constrained the creation of distributional maps, impeding effective vector control. This limitation presents a significant challenge in the fight against malaria in Nigeria through vector control [10]. The utility of species distribution models and their outputs offer a valuable approach to address knowledge gaps in situations where there is a lack of data. They can indicate areas of suitable habitat for each species where data is lacking, enabling evidence-based estimates of the risk of malaria transmission in regions not covered by current interventions [10,16].

An ecological niche is defined as the range of natural conditions (biotic and abiotic variables) where a species can maintain viable population sizes without migration [17]. Ecological Niche Modeling serves as a valuable tool that extracts and delineates the fundamental boundaries of a species’ niche at each occurrence location within a chosen dataset, considering a set of relevant environmental covariates. The predictions generated by Ecological Niche Modeling can fill gaps resulting from data shortages in surveillance [17,18]. Analysis on the diversity of *An. gambiae* s.l. in Nigeria have been published elsewhere [10], and this study focused on understanding the spatial distribution and potential spread of the non-*gambiae* species in Nigeria. This study models the spatial distribution of secondary malaria vectors in 20 states across Nigeria and presents a species distribution model to address information gaps, offering evidence-based outputs for public health decision-makers to guide future national surveillance and control programs.

## Materials and methods

### Study area

Nigeria (9.0820° N, 8.6753° E) has 36 states plus FCT with an estimated population of 215,266,57728. Generally, the climate in the country is typically tropical with two distinct seasons, rainy (May to October) and dry (November to April). The mean annual weather conditions in the country range from 24.0 °C to 30.20 °C (Temperature), 31.1–85% (Relative Humidity) and 314 mm–1871 mm (Rainfall) [10]. In terms of vegetation, the states in the north consist of Guinea, Sudan and Sahel Savannah while the southern part includes the mangrove and the forest in addition to the Guinea Savannah [10]. The main method of vector control in the study areas is insecticide-treated bed nets (ITNs). In the past 5 years, more ITNs have been deployed in the states through free mass distributions, resulting into high coverage (over 70%) across the study areas [10].

### Mosquito collection and identification

**Fig 1:**
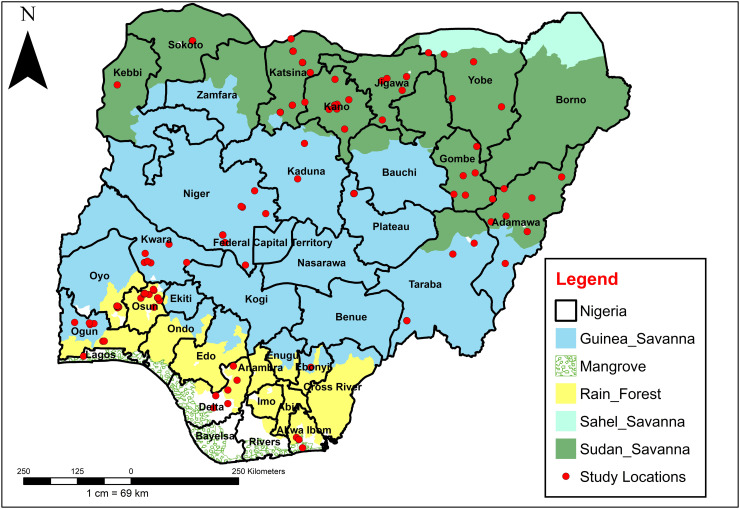
Map of Nigeria showing the sentinel sites from the 20 selected states for *Anopheles* surveillance. This figure was created by the authors in R programming software (R version 4.1.2, Vienna, Austria). Available at https://www.R-project.org/. The Nigerian shapefile was obtained from World Bank Data Catalog (an Open license standardized resource of boundaries (i.e., state, county) for every country in the world).

Adult (using both CDC light traps and pyrethrum spray catches (PSC)) and larval samples of mosquitoes were collected in twenty states (Fig 1) which span all the ecological zones in the country (Fig 1). The World Health Organization (WHO) and Center for Disease Control (CDC)’s entomological protocols [19] for field and laboratory studies on malaria vectors were employed in carrying out the studies. Adult mosquitoes were uniformly collected on monthly basis from the year 2020 to 2022 using both PSC and CDC light traps (see S1 File for full details). Surveys of larval breeding sites were also carried out in 120 LGAs across the 20 States. *Anopheles* larvae were collected from identified breeding sites such as permanent water bodies, rice fields, small water pools, or impoundments, using a dipping method described by WHO [19] and the location of all sites were georeferenced using a GPS device. Collected larvae and adult mosquitoes were transported to the insectaries (for each state). The larvae reared to adult under ambient laboratory conditions and the emerged adults were identified morphologically by trains personnels and finally validated by the Principal Investigator (for each state) using the keys of Gilles and Coetzee [20] under ×  1000 Dino-lite HD color CMOS sensor high speed digital microscope (model number, AD4113T-12 V). After identification, the mosquitoes were sorted into various Anopheline species. The collected mosquitoes were also identified using diagnostic PCR (Details has been published elsewhere [10,21]). Routine data collection for Akwa Ibom, Ebonyi, Oyo, Plateau, Sokoto, and Kebbi States was supported by the U.S. President’s Malaria Initiative and made available through a data-sharing arrangement between the NMEP, NIMR, PMI VectorLink, and the Evolve project.

### Environmental data

Climatic variables such as temperature and precipitation influence species distribution at global and meso-scales, topographic variables such as altitude and aspect have more influence at meso and topo-scales whereas land-cover variables such as percent canopy cover can influence distributions at the micro-scale [22]. Hence, climatic and topographic level variables were used here to predict the distribution of the other Anopheline species (non-*gambiae*).

We considered 19 environmental and four topographical variables as potential predictors of the target species habitat distribution [17,23,24]. These variables were chosen based on their biological relevance to the target species distributions [25–28]. The nineteen bioclimatic variables with a 2.5 min spatial resolution (about. 1 km^2^) were downloaded from the WorldClim database (http://www.worldclim.org/) [29]. Elevation data 1 km^2^- resolution was obtained from the Shuttle Radar Topography Mission (SRTM). The elevation data was used to generate slope, aspect, and hillshade (all in degrees) using the Spatial Analyst tool/surface in using ArcGIS 10.7.1 software. The coordinates for all presence and absence data were taken in decimal degrees (to four decimal places) and plotted using Google Earth to check for annotation errors. After downloading the climatic files, the Nigeria layer was extracted by using a boundary mask.

All combinations of the 23 environmental and topographic variables have been tested for multi-collinearity through the calculation of R-squared in linear regression analysis in R software ver. 4.1.2. Since some of these bioclimatic variables were strongly correlated (R2 ≥  0.7), only those variables that demonstrated little correlation with other predictors were retained [10]. Finally, a total of 11 environmental and topographical variables were selected in this study (R2 <  0.7) (Table 1).

**Table 1:** Environmental variables used for modeling the potential distribution of Anopheline species (non-*gambiae*) In the present study.

No	Variable	Code/Unit	Source
1	Annual mean temperature	Bio1(°C)	WorldClim
2	Mean Diurnal Range (Mean of monthly (max temp—min temp))	Bio2(°C)	WorldClim
3	Isothermality	Bio3(°C)	WorldClim
4	Mean Temperature of Driest Quarter	Bio9(°C)	WorldClim
5	Precipitation of the Wettest Month	Bio13(mm)	WorldClim
6	Precipitation of the Driest Month	Bio14(mm)	WorldClim
7	Precipitation of the Coldest Quarter	Bio19(mm)	WorldClim
8	Precipitation of Coldest Quarter	Bio19(mm)	WorldClim
9	Slope	–	SRTM
10	Aspect	–	SRTM
11	Hillshade	–	SRTM

### Modelling

To model the distribution of the target species and predict its presence probability and abundance, we employed a Random Forest (RF) algorithm, a widely-used ensemble machine learning technique known for its robust performance in handling complex relationships between predictor variables and response variables [30]. The analysis was conducted in R (R Foundation for Statistical Computing, Vienna, Austria)), with the ‘dismo’, ‘raster’, ‘pROC’, and ‘RadomForest’ packages. For the study area, the analysis requires both species presence and absence data and environmental variable (continuous or categorical) layers. Before model construction, the predictor variables were standardized, using the scale function to have a mean of 0 and a standard deviation of 1. This scaling step was essential to ensure that all predictors contributed equally to the model and to prevent any undue influence from variables with large scales.

To model presence/absence, we trained a Random Forest regression model using the standardized predictor variables and a binary response variable, indicating whether the species of interest (non-*gambiae* spp.) is present (1) or absent (0). The trained model was then used to generate predictions, providing estimates of the probability of species presence, ranging from 0 (“unsuitable”) to 0.99 (“best habitat suitability”) [10]. The Random Forest model was trained on 500 decision trees, and its performance was assessed by applying cross-validation techniques. In parallel, a separate Random Forest model was trained to predict species abundance across the study area [30], by examining the relationship between the predictors (environmental variables) and the yearly average abundance of each mosquito species over the three-year period. To further investigate the relationship between each environmental predictor and the probability of species presence, response curves for each predictor variable was generated. These response curves illustrate how changes in the predictor values influence the predicted probability of species occurrence.

A total of 75% of the location point data were used for training, and the remaining 25% were used for testing the predictive ability of the model (Model validation), in addition 10 replicates were considered. The output prediction raster for the average models were loaded in ArcGIS 10.7.1 where habitat suitability was divided into 4 classes; Not suitable (0–0.2), low ( > 0.2–0.4), moderate ( > 0.4–0.6), high ( > 0.6) using natural breaks in the symbology tools to produce the habitat suitability model picture [10].

### Model performance evaluation

To assess the performance of the presence/absence model, we performed Receiver Operating Characteristic (ROC) analysis using the ‘pROC’ package. The ROC analysis evaluated the model’s sensitivity and specificity, and we computed the Area Under the Curve (AUC) as a measure of model accuracy [10,30]. A higher AUC value indicates better discriminatory power (AUC values 0.5 =  random to 1 =  perfect discrimination) of the model in distinguishing between presence and absence points. We also computed the root mean square error (RMSE) and the percentage of variation explained by the model. Both parameters estimate model accuracy and fitness, indicating how well the model fits the data. Additionally, we calculated the percentage increase in mean square error (%IncMSE) and node purity (IncNodePurity) to assess variable importance for the models [30].

### Ethical approval

Ethical clearance for this study was obtained from the Ethics review committee, Federal Ministry of Health. All methods including mosquito larva collection and breeding, laboratory analysis and data management were performed in accordance with the 1964 Declarations of Helsinki.

## Results

### Spatial distribution of Anopheline species collected from the 20 states

**Fig 2:**
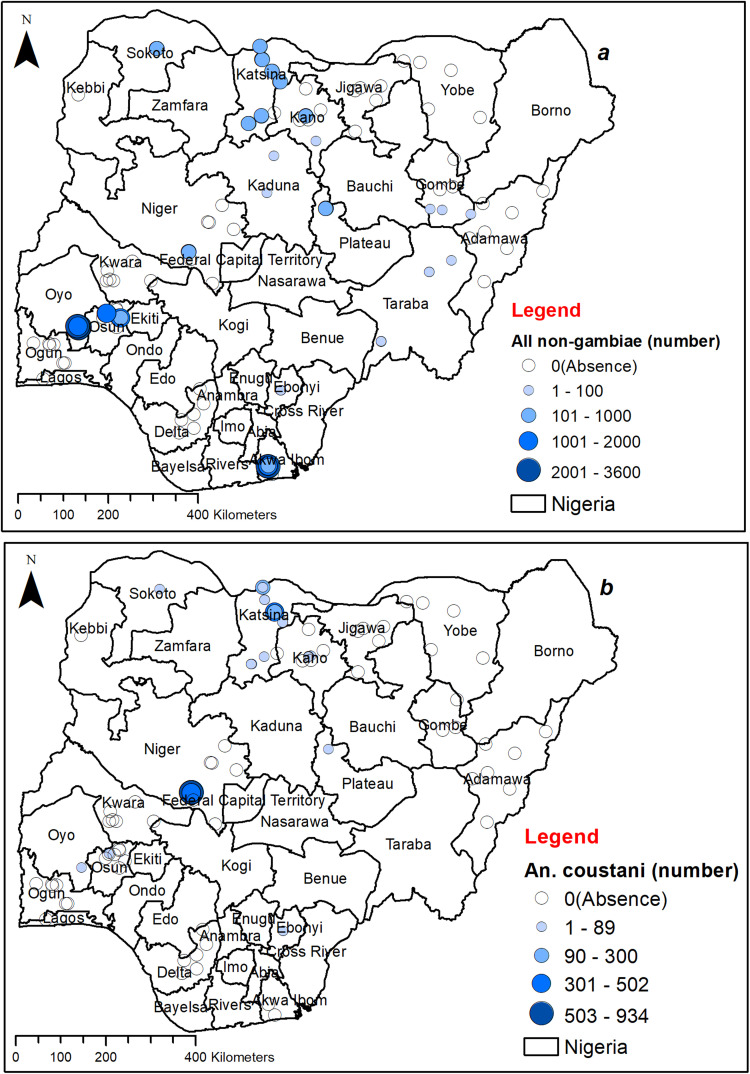
Spatial distribution of *An.* non-*gambiae* species collected. (a) Distribution of all species in Nigeria. (b) Distribution of *An. coustani* from the collection sites. This figure was created by the authors in R programming software (R version 4.1.2, Vienna, Austria). Available at https://www.R-project.org/. The Nigerian shapefile was obtained from World Bank Data Catalog (an Open license standardized resource of boundaries (i.e., state, county) for every country in the world.

*Anopheline* species other than *An*. *gambiae* complex (non-*gambiae* species) were found in only 10 out of the 20 states, in various proportions (Fig 2a). However, members of the *An*. *gambiae* s.l. were found in all locations including the remaining locations where other species were found. Generally, non-*gambiae* species were widely distributed across the country, with more occurrence locations in the northern part, where the number of mosquitoes caught ranged from less than 100 to 3600 per year. In terms of abundance, their population seems to be higher in the southern part of the country, notably in Oyo, Osun, and Akwa-Ibom (Fig 2a)

**Fig 3:**
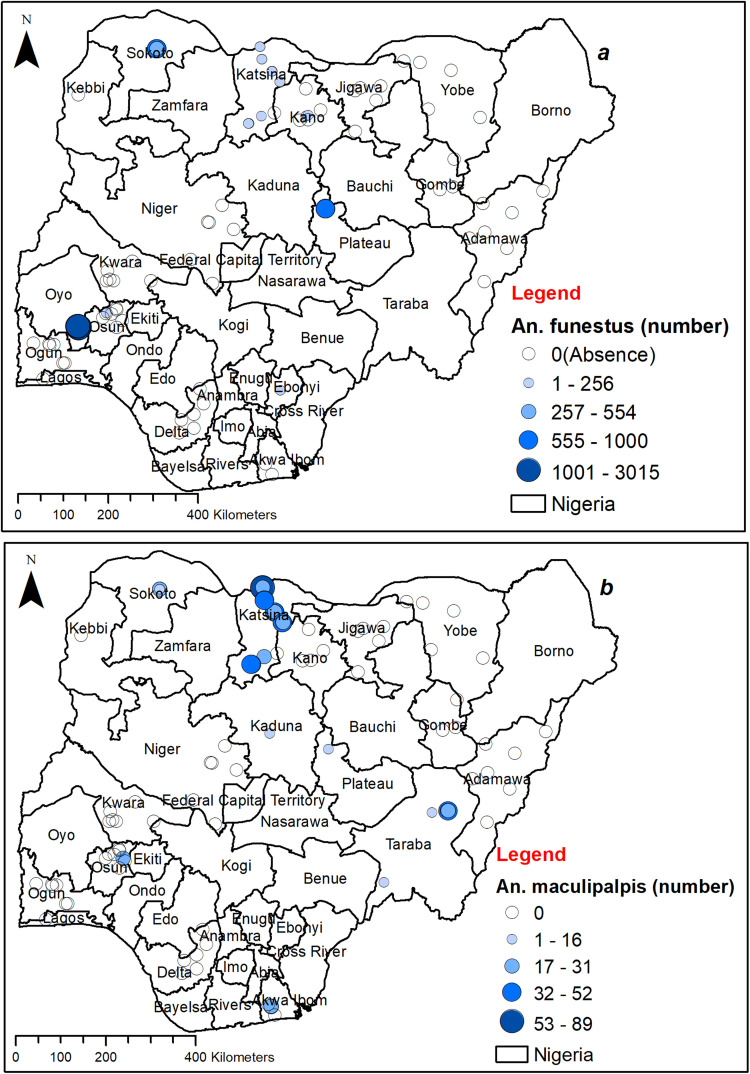
Spatial distribution of *An.* non-*gambiae* species collected. (a) Distribution of *An. funestus* in Nigeria. (b) Distribution of *An. maculipalpis* in Nigeria. This figure was created by the authors in R programming software (R version 4.1.2, Vienna, Austria). Available at https://www.R-project.org/. The Nigerian shapefile was obtained from World Bank Data Catalog (an Open license standardized resource of boundaries (i.e., state, county) for every country in the world.

**Fig 4:**
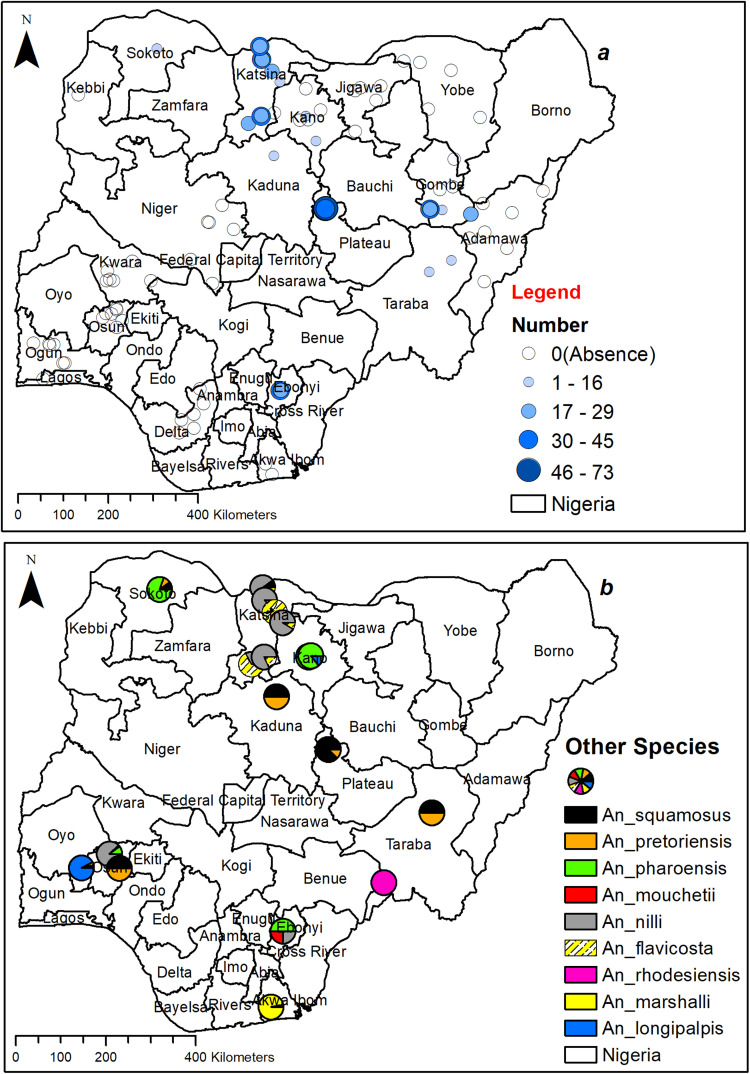
Spatial distribution of *An.* non-*gambiae* species collected. (a) Distribution of *An. rufipes* in Nigeria. (b) Distribution of other *An.* non-*gambiae* spp in Nigeria. This figure was created by the authors in R programming software (R version 4.1.2, Vienna, Austria). Available at https://www.R-project.org/. The Nigerian shapefile was obtained from World Bank Data Catalog (an Open license standardized resource of boundaries (i.e., state, county) for every country in the world.

*An. coustani* and *An. funestus* were identified in limited locations in Oyo, Osun, Niger, and Plateau States, while being more present in more sites in the northern part of the country. The number captured ranged from fewer than 89 to 934 per year for *An. coustani* (Fig 2b) and less than 256 to 3015 annually for *An. funestus* (Fig 3a). *An. coustani* exhibited a higher population in Niger and certain parts of Katsina state in comparison to other locations (Fig 2b), while *An. funestus* had a relatively higher numbers in Oyo, followed by Plateau and Sokoto, with lower numbers in many northern regions (Fig 3a). Conversely, *An. maculipalpis* was found in limited locations in Osun and Akwa Ibom but was more widespread in the North, with capture numbers ranging from fewer than 16 to 89 per year (Fig 3b). *An. maculipalpis* were more captured in the northern parts than the southern regions. Similarly, the number of *An. rufipes* caught was generally low, ranging from fewer than 16 to 73 mosquitoes per year (Fig 4a), with a higher capture number in the north. *An. rufipes* was only found in the south, specifically in Ebonyi (Fig 4a).

Other non-*gambiae* species found during this study include *An. squamosus, An. pretoriensis, An. pharoensis, An. mouchetii, An. nili, An. flavicosta, An. rhodesiensis, An. marshalii,* and *An. longipalpis*. These mosquitoes were found in sympatry throughout the country, except in Kano and Taraba, where only one species was observed (Fig 4b). Further details on the number of non-*gambiae Anopheles* species caught each year, categorized by species and trapping method, as well as monthly catches, are presented in S2–S4 Files.

### Potential habitat distribution for Anopheline other than An. *gambiae* complex (non-*gambiae* species) in Nigeria

**Fig 5:**
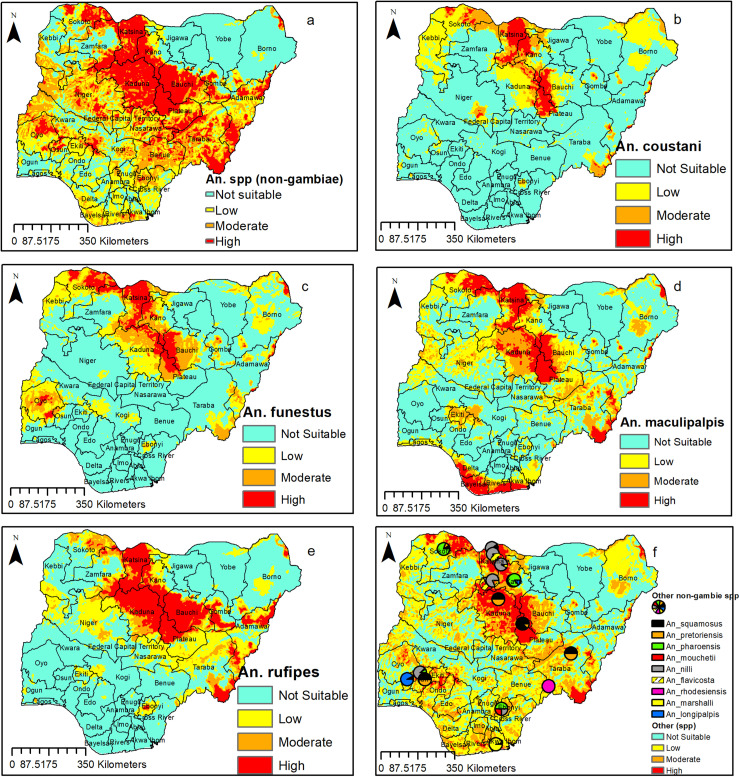
Predicted distribution of *Anopheles* (non-*gambiae*) species in Nigeria. (a) All non-*gambiae* species (b) *An. coustani* (c) *An. funestus* (d) *An. maculipalpis* (e) *An. rufipes* (f) Other non *gambiae* species. This figure was created by the authors in R programming software (R version 4.1.2, Vienna, Austria). Available at https://www.R-project.org/. The Nigerian shapefile was obtained from World Bank Data Catalog (an Open license standardized resource of boundaries (i.e., state, county) for every country in the world.

Maps for the potential distribution of the non-*gambiae* species, are presented in Fig 5. The results showed widespread habitat suitability for these species across the country. Only three states Yobe, Jigawa and Borno seems to have large areas that are not suitable for these species (Fig 5a). The habitat suitability for different *An.* non-*gambiae* species are highlighted below;

Our model predicted high suitability for *An. coustani* in larger parts of 6 states (Sokoto, Katsina, Kano, Plateau, Bauchi and Kaduna). On the other hand, few parts of Niger, Ebonyi, Taraba, Gombe, Borno, Jigawa, and Adamawa were highly suitable for *An. coustani, while* some parts of Ogun, Oyo, Kebbi, Yobe, Borno, and Taraba had low to moderate habitat suitability for this species (Fig 5b)*.* All parts of Ondo, Edo, and south-eastern part of the country (except for Ebonyi) seems to be unsuitable for *An. coustani* (Fig 5b).

*An. funestus* has more range expansion compared with *An. coustani*. Large parts of 9 states (Oyo, Sokoto, Zamfara, Kaduna, Kano, Katsina, Bauchi, Plateau, and Gombe) were highly suitable for *An. funestus*. On the other hand, few parts of Borno, Adamawa, Yobe and Osun seems to be highly suitable for *An. funestus,* while some parts of Kwara, Ogun, Kogi, Ebonyi, Kebbi, Niger, Taraba, FCT, Ekiti, and Osun showed between low to moderate suitability for *An. funestus* (Fig 5c).

For *An. maculipalpis,* our model predicted a large part of 12 states (Sokoto, Katsina, Kaduna, Kano, Plateau, Bauchi, Adamawa, Taraba, Delta, Bayelsa, Rivers and Akwa Ibom) to be highly suitable. In addition, some parts of Borno, Cross River, Ekiti, Ondo, Niger, Nasarawa, Kebbi, and FCT seems to be highly suitable for *An. maculipalpis* (Fig 5d) where large parts of Lagos, Benue, Cross River, Ebonyi, Edo, Ondo showed between low to moderate suitability for *An. maculipalpis* (Fig 5d).

For *An. rufipes,* our model predicted a large part of 11 states (Sokoto, Zamfara, Katsina, Kaduna, Kano, Bauchi, Plateau, Gombe, Adamawa, Taraba, and Ebonyi) to be highly suitable. Also, some parts of Niger, Nasarawa, FCT, Kebbi, and Borno seems to be highly suitable for *An. An. rufipes* (Fig 5e) where large parts of Niger, Ekiti, Kwara, Kogi, Benue, and some parts of Ogun, Oyo, Lagos, Edo and Cross Rivers showed between low to moderate suitability for *An. rufipes* (Fig 5e).

The detection of other species apart from *An. funestus*, *An. coustani*, *An. maculipalpis*, and *An. rufipes*, in very few locations across the country resulted in our inability to model for each of the species. As a result, they were represented as point maps which showed their presence across the northern and Southern parts of the country (Fig 5f).

### Modeled relative abundance for Anopheline other than An. *gambiae* complex (non-*gambiae* species) in Nigeria

**Fig 6:**
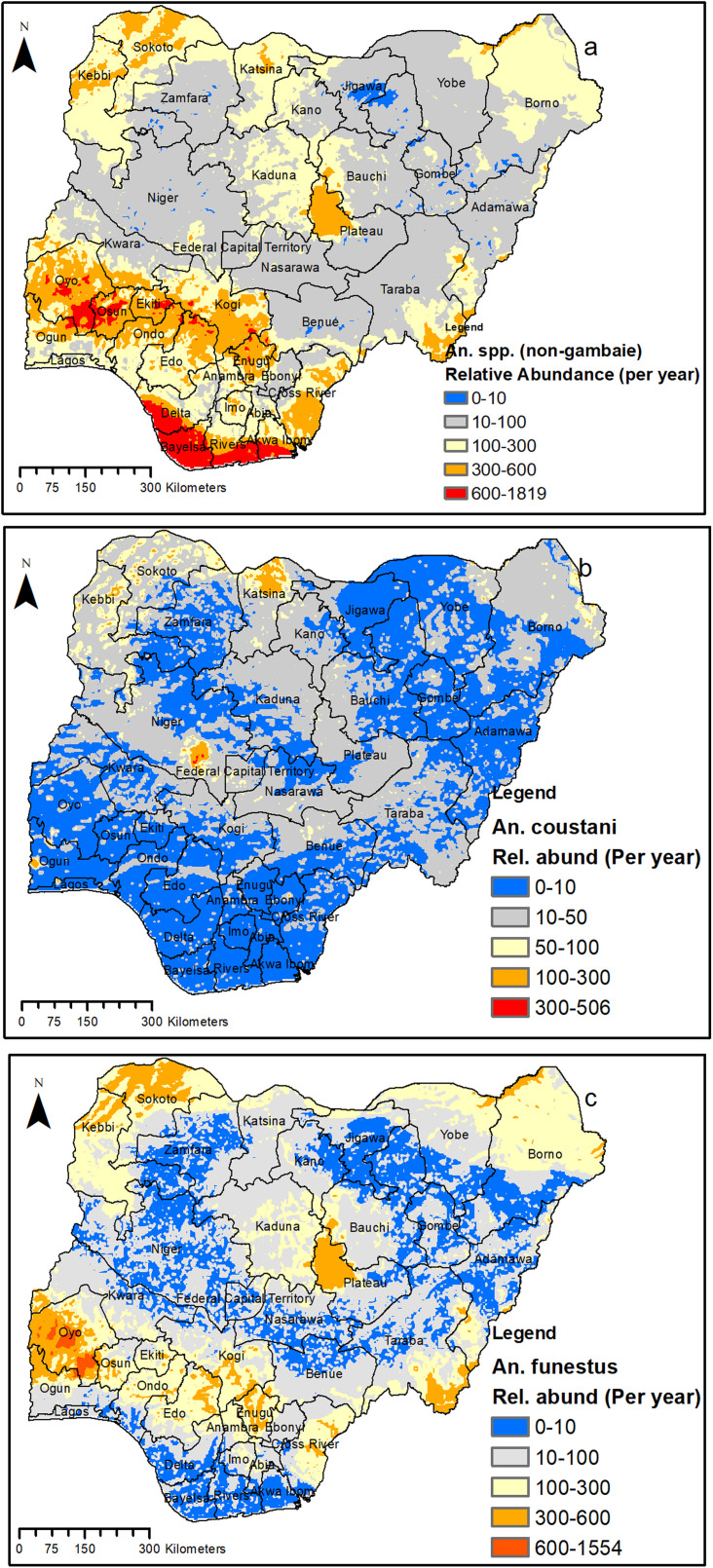
Predicted abundance of *Anopheles* non-*gambiae* species in Nigeria. (a) All non-*gambiae* species (b) *An. coustani* (c) *An. funestus*. This figure was created by the authors in R programming software (R version 4.1.2, Vienna, Austria). Available at https://www.R-project.org/. The Nigerian shapefile was obtained from World Bank Data Catalog (an Open license standardized resource of boundaries (i.e., state, county) for every country in the world.

**Fig 7:**
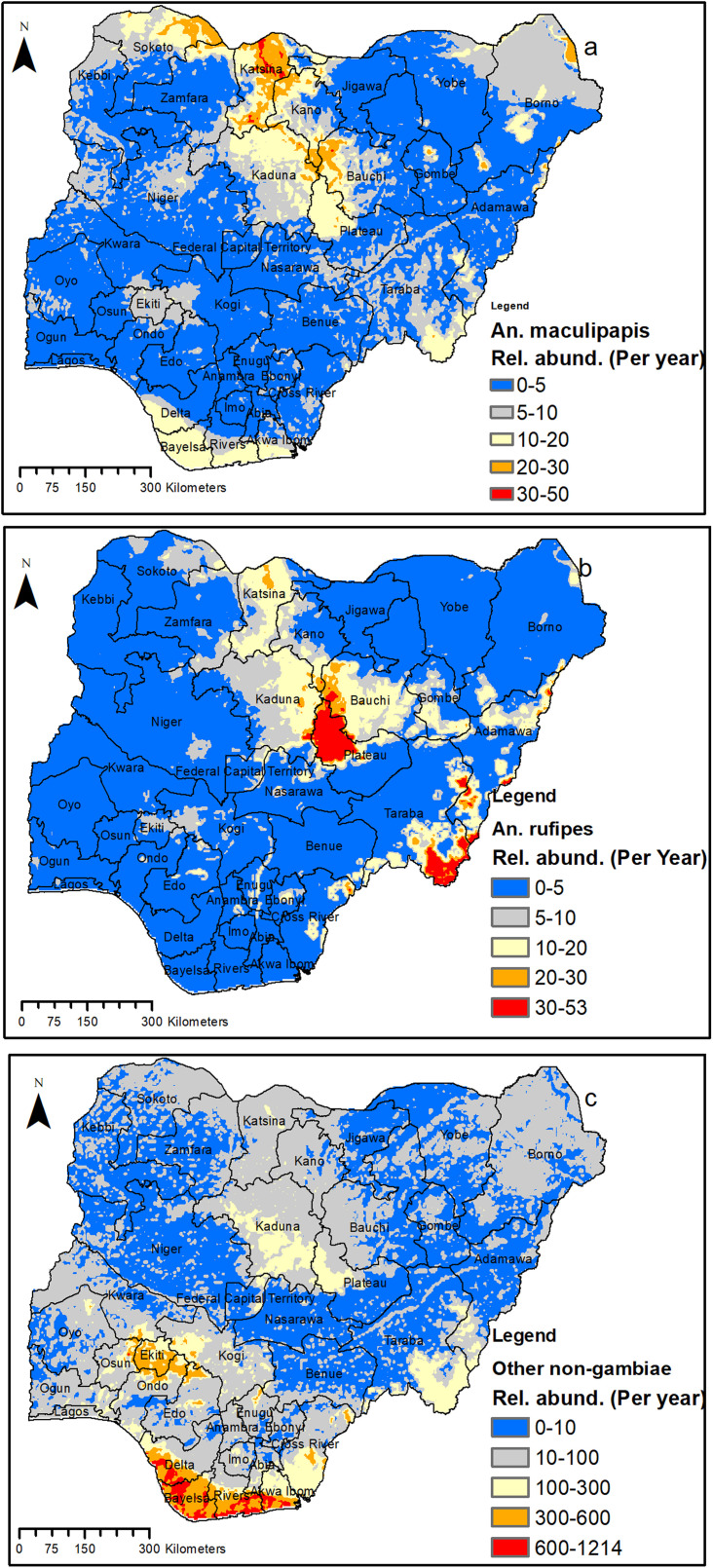
Predicted abundance of *Anopheles* (non-*gambiae*) species in Nigeria. (a) *An*. *maculipalpis* (b) *An*. *rufipes* (c) Other non *gambiae* species. This figure was created by the authors in R programming software (R version 4.1.2, Vienna, Austria). Available at https://www.R-project.org/. The Nigerian shapefile was obtained from World Bank Data Catalog (an Open license standardized resource of boundaries (i.e., state, county) for every country in the world.

The model output for the relative abundance of the non-*gambiae* species is presented in Figs 6 and 7. The results showed a relatively high population density (600–1819) per year for all the non-*gambiae* species in the southern part as compared to the north part (Fig 6a). The modeled relative abundance for different *An*. non-*gambiae* species is highlighted below:

Our model predicted high density of *An. coustani* in some parts of Ogun, Niger, Kebbi, Sokoto, and Katsina, while the rest of the country has a relatively lower density for this species (Fig 6b). For *An. funestus*, our model predicted larger areas with a high population density compared to that of *An. coustani*. Large parts of eight states (Oyo, Kebbi, Sokoto, Plateau, Taraba, Kogi, Enugu, and Edo), along with some parts of another nine states (Ogun, Osun, Ondo, Cross Rivers, Adamawa, Kaduna, Bauchi, Yobe, and Borno), were predicted to have a high density for *An. funestus* (Fig 6c).

On the other hand, a few states, mainly in the northern part of the country, were relatively predicted to have a high population density (although their population is predicted to be very small) for *An. maculipalpis* (Fig 7a) and *An. rufipes* (Fig 7b) compared with other states in the country. For *An. maculipalpis*, some areas of Sokoto, Katsina, Kano, and Bauchi were predicted to have a relatively higher population density compared to other states, while larger areas of Plateau, Taraba, and Adamawa were predicted to have a relatively higher density for *An. rufipes*. The relative abundance of other remaining species (Fig 7c) indicated high population density mainly in the southern part of the Country. These includes Ekiti, Ondo, Delta, Bayelsa, Rivers and Akwa Ibom (Fig 7c).

### Model performance and influencing factors

**Fig 8:**
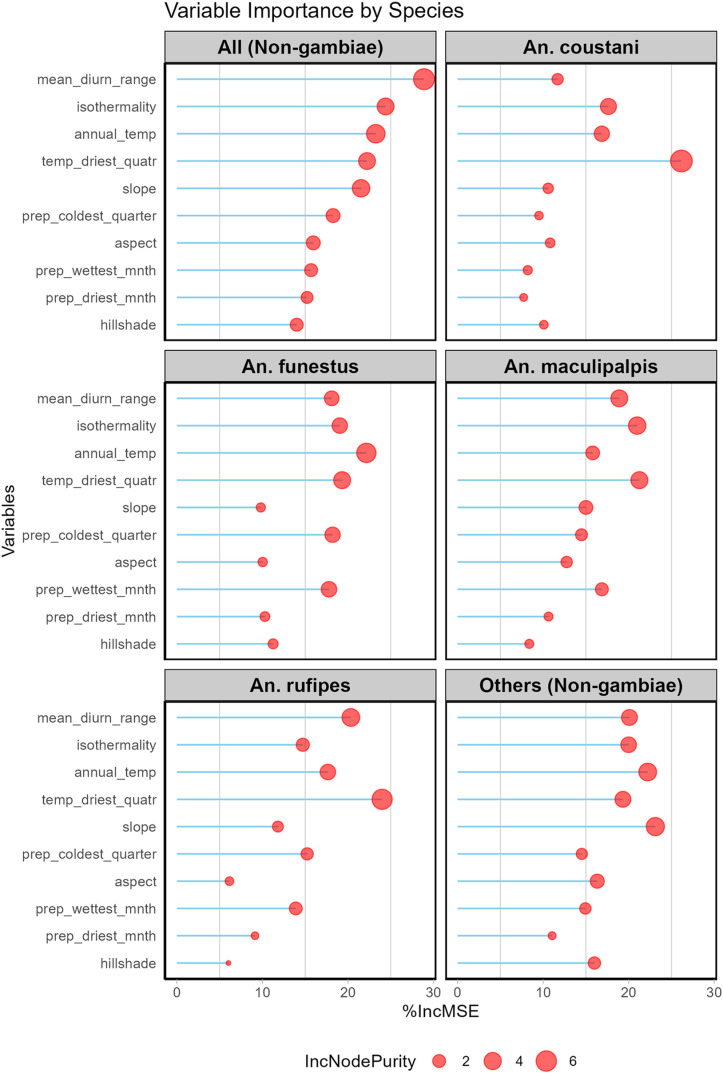
Level of importance of the variables used in the modeling of *the Anopheles (non-gambiae) species* distribution. Light blue line connotes %IncMSE while blue circle or dots connotes IncNodePurity. The longer the blue line and the bigger the circle the more important the variable contribution to the model.

**Fig 9:**
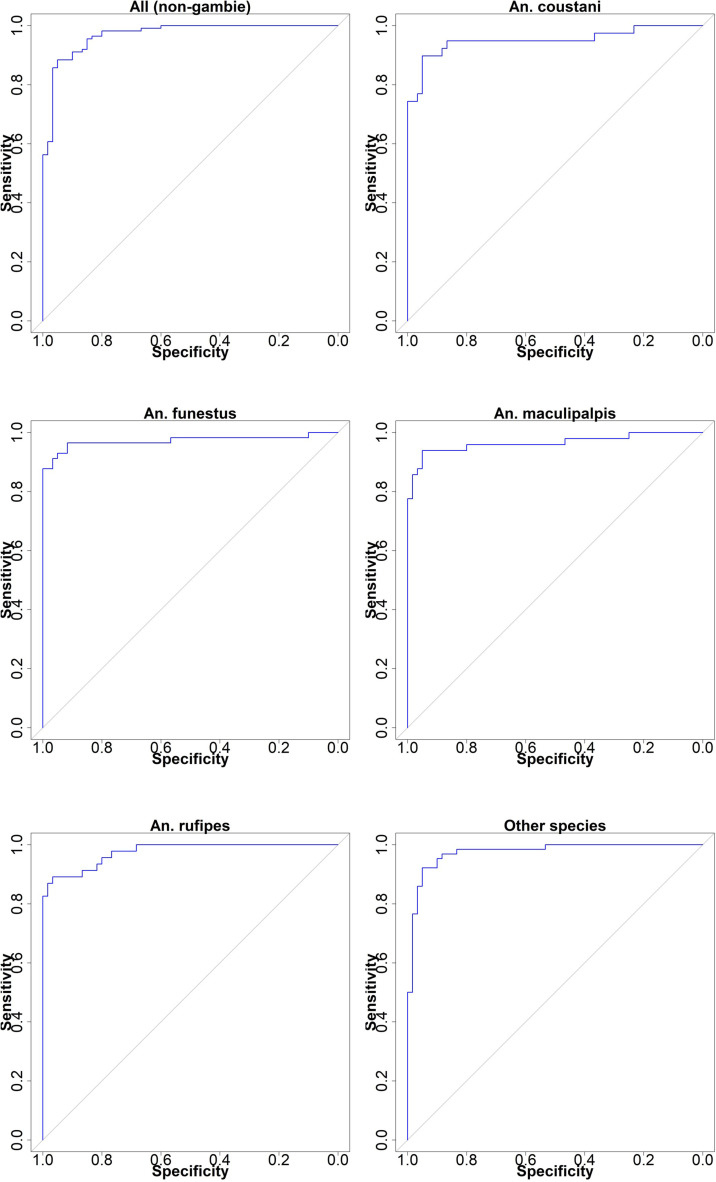
Area under the curve (AUC) for all the *Anopheles* (non-*gambiae*) species. Blue line indicates the mean value for 10 random forest replicate runs.

The average percent increase in mean square error (%IncMSE) and increase in node purity (IncNodePurity) of the 11 variables used in modeling the distribution of An. non-*gambiae* species in this study were presented in Fig 8. The ROC curve is shown in Fig 9. The average value of the AUC for the 10 replicate runs was not less than 0.9 for all the models. Furthermore, the mean squared residuals (MSR) and the percentage of variation explained by the models are approximately 0.1 and greater than 70%, respectively, for all the models (Table 2). These values show an excellent performance of the models, as an AUC value greater than 0.8 indicate higher sensitivity and specificity, and an MSR of less than 0.1 indicates excellent model fits, thereby increasing the confidence in the models. The variables importance and response curves of each model is highlighted below;

**Table 2:** Model performance characteristics.

Variables	*An.* (non-*gambiae*) species					
	*All*	*An.* *coustani*	*An.* *funestus*	*An.* *maculipalpis*	*An.* *rufipes*	*Other* *species*
Mean of squared residuals	0.0632	0.0715	0.0597	0.0533	0.0642	0.0720
AUC values	0.97	0.94	0.98	0.98	0.97	0.97
% of variations explained (%)	71.99	70.31	76.13	78.44	73.83	71.09

### All *An*. (non-*gambiae*) species

**Fig 10:**
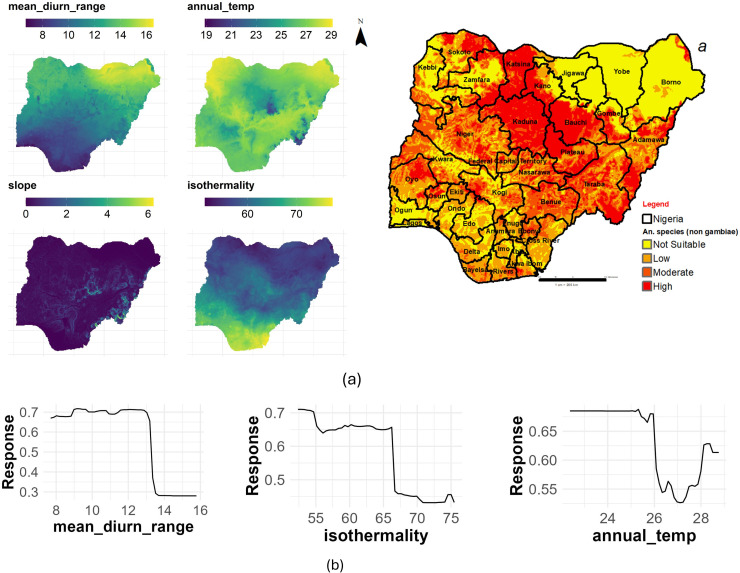
Estimates of the highest contributing variables that determines the geographical distribution of *An*. non *gambiae* species. (a) The highest environmental variables that estimate to control the geographical distribution in Nigeria. Variable contributions (mean diurnal range, isothermality and mean annual temperature), (b) Response curves of three environmental predictors used in Random Forest model for An. non-*gambiae* species. This figure was created by the authors in R programming software (R version 4.1.2, Vienna, Austria). Available at https://www.R-project.org/. The Nigerian shapefile was obtained from World Bank Data Catalog (an Open license standardized resource of boundaries (i.e., state, county) for every country in the world.

The mean diurnal range had the highest contribution with %IncMSE of 28.89, followed by isothermality (%IncMSE of 24.40) and annual mean temperature (%IncMSE of 23.35). The results showed that these variables are strong predictors of all *An.* non-*gambiae* species distribution in Nigeria (Table 3). Fig 10 shows the main highest estimated environmental variables (contributions) that determine the distribution of all *An.* non-*gambiae* in Nigeria. Our spatial analysis of the entire country revealed that the mean diurnal range ranged from 7 to 16.3 °C, isothermality ranged from 54 to 76%, and annual mean temperature ranged from 18.9 to 29.1 °C (Fig 10a). The response curves of these three variables to the habitat suitability of all *Anopheles* non-*gambiae* species are shown in Fig 10b. These curves indicate that a mean diurnal range between 7 and 13 °C favors the potential distribution of all *Anopheline* (non-*gambiae*) species. Similarly, isothermality values between 54 and 65% significantly favor their distribution, as does an annual mean temperature range of 22 to 25.8 °C (Fig 10b).

**Table 3:** Variable importance ranking using the %IncMSE values for all the predicted *Anopheles species.*

Variables	% increase in Mean Squared Error					
	*All*	*An.* *coustani*	*An.* *funestus*	*An.* *maculipalpis*	*An.* *rufipes*	*Other* *species*
Annual Mean Temperature	**23.35** ^**c**^	**16.86** ^**c**^	**22.15** ^**a**^	15.79^e^	**17.65** ^**c**^	**22.22** ^**b**^
Mean Diurnal Range	**28.89** ^**a**^	11.69^d^	18.10^e^	**18.90** ^**c**^	**20.34** ^**b**^	**20.09** ^**c**^
Isothermality	**24.40** ^**b**^	**17.63** ^**b**^	**19.04** ^**c**^	**20.99** ^**b**^	14.72^e^	19.99^d^
Precipitation of the Wettest Month	15.69^h^	8.20^i^	17.78^f^	16.86^d^	13.90^f^	14.93^h^
Precipitation of the Driest Month	15.22^i^	7.72^j^	10.29^h^	10.63^1^	9.13^h^	11.05^j^
Precipitation of the Coldest Quarter	18.26^f^	9.51^h^	18.20^d^	14.48^g^	15.23^d^	14.53^i^
Mean Temperature of Driest Quarter	22.23^d^	**26.16** ^**a**^	**19.32** ^**b**^	**21.25** ^**a**^	**23.99** ^**a**^	19.32^e^
Aspect	15.94^g^	10.82^e^	10.03^1^	12.75^h^	6.15^i^	16.32^f^
Slope	21.53^e^	10.59^f^	9.81^j^	15.00^f^	11.81^g^	**23.11** ^**a**^
Hillshade	14.01^j^	10.08^g^	11.25^g^	8.38^j^	6.03^j^	15.99^g^

The superscripts (alphabets) in a column, connotes variable importance ranking in descending order for each model (species) using the %IncMSE values.

### An. coustani

**Fig 11:**
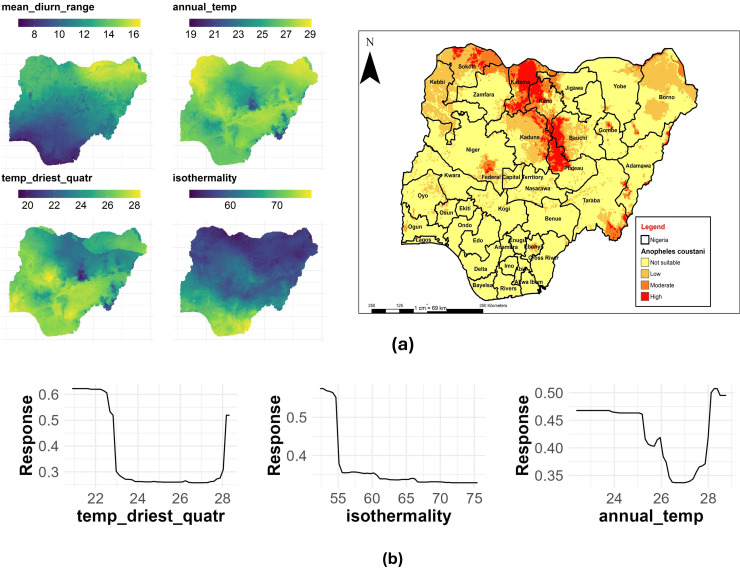
Estimates of the highest contributing variables that determines the geographical distribution of *An. coustani.* (a) The highest environmental variables that estimate to control the geographical distribution of An. coustani in Nigeria. Variable contributions (temperature of driest quarter, mean annual temperature and isothermality), (b) response curves of three environmental predictors used in Random Forest model for *An. coustani*. This figure was created by the authors in R programming software (R version 4.1.2, Vienna, Austria). Available at https://www.R-project.org/. The Nigerian shapefile was obtained from World Bank Data Catalog (an Open license standardized resource of boundaries (i.e., state, county) for every country in the world.

The temperature of the driest quarter had the highest contribution to this species distribution, with a %IncMSE of 26.16, followed by isothermality (%IncMSE of 17.63) and annual mean temperature (%IncMSE of 16.86) (Table 3). Fig 11 shows the main highest estimated environmental variables (contributions) that determine the distribution of *An. coustani* in Nigeria. We found out that the temperature of the driest quarter ranged from 18.3 to 28.4 °C, isothermality ranged from 54 to 76 °C, while annual mean temperature ranged from 18.9 to 29.1 °C (Fig 11a). The response curves of the three variables to *An. coustani* habitat suitability are shown in Fig. 11b. These curves demonstrate that a temperature of the driest quarter between 20 and 22 °C favors the potential distribution of *An. coustani*. Similarly, an isothermality value of < 55 °C significantly and potentially favored their distribution, while an annual mean temperature ranging between 28 and 29 °C significantly favored the distribution of *An. coustani* in Nigeria (Fig 11b)

### An. funestus

**Fig 12:**
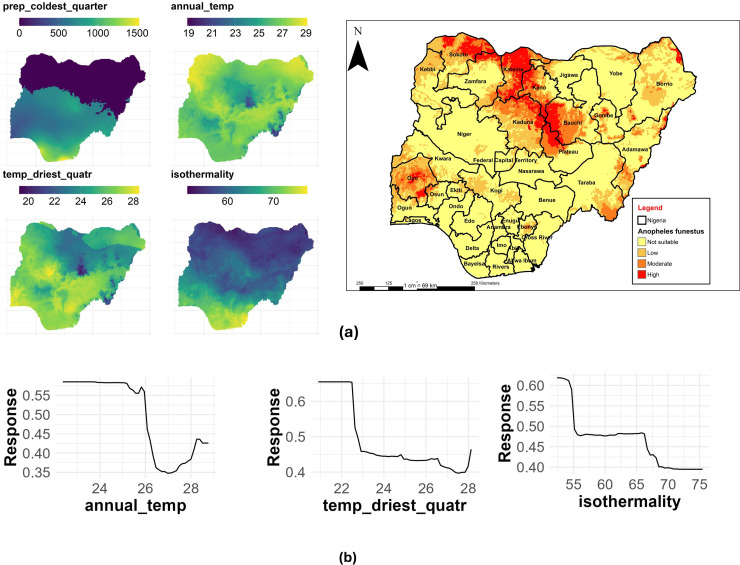
Estimates of the highest contributing variables that determines the geographical distribution of *An. funestus.* (a) The highest environmental variables that estimate to control the geographical distribution of An. funestus in Nigeria. Variable contributions (mean annual temperature, temperature of driest quarter, and precipitation of wettest month), (b) response curves of three environmental predictors used in Random Forest model for *An. funestus*. This figure was created by the authors in R programming software (R version 4.1.2, Vienna, Austria). Available at https://www.R-project.org/. The Nigerian shapefile was obtained from World Bank Data Catalog (an Open license standardized resource of boundaries (i.e., state, county) for every country in the world.

The annual mean temperature had the highest contribution with a %IncMSE of 22.15, followed by the mean temperature of the driest quarter (%IncMSE of 19.32) and Isothermality (%IncMSE of 19.04) to the distribution of *An. funestus* in Nigeria (Table 3). Fig 12 shows the main highest estimated environmental variables (contributions) that determine the distribution of *An. funestus* in Nigeria. We found out that the annual mean temperature ranged from 18.9 to 29.1 °C, the temperature of the driest quarter ranged from 18.3 to 28.4°C, while isothermality ranged from 54 to 76 °C (Fig 12a). The response curves of the three variables to *An. funestus* habitat suitability are shown in Fig 12b. These curves demonstrate that an annual temperature between 22 and 24.8°C favors the potential distribution of *An. funestus*. Similarly, the temperature of the driest quarter ranging between 20 to 22 °C significantly and potentially favored their distribution with an isothermality of < 55°C (Fig 12b).

### An. maculipalpis

**Fig 13:**
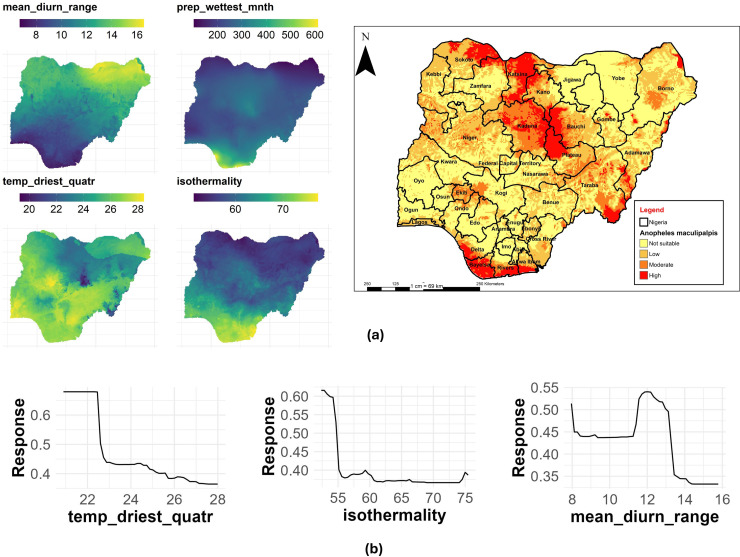
Estimates of the highest contributing variables that determines the geographical distribution of *An. maculipalpis.* (a) The highest environmental variables that estimate to control the geographical distribution of An. maculipalpis in Nigeria. Variable contributions (temperature of driest quarter, isothermality and mean diurnal range), (b) Response curves of three environmental predictors used in Random Forest model for *An. maculipalpis*. This figure was created by the authors in R programming software (R version 4.1.2, Vienna, Austria). Available at https://www.R-project.org/. The Nigerian shapefile was obtained from World Bank Data Catalog (an Open license standardized resource of boundaries (i.e., state, county) for every country in the world.

The mean temperature of the driest quarter had the highest contribution with a %IncMSE of 21.25, followed by isothermality (%IncMSE of 20.99) and mean diurnal range (%IncMSE of 18.90) to the distribution of *An. maculipalpis* in Nigeria (Table 3). Fig 13 shows the main highest estimated environmental variables (contributions) that determine the distribution of *An. maculipalpis* in Nigeria. We found out that the mean temperature of the driest quarter ranged from 18.3 to 28.4 °C, isothermality ranged from 54 to 76 °C, while mean diurnal range ranged from 7 to 16.3 °C, (Fig 13a). The response curves of the three variables to *An. maculipalpis* habitat suitability are shown in Fig 13b. These curves demonstrate that the temperature of the driest quarter ranging between 20 to 22 °C favors the potential distribution of *An. maculipalpis*. Similarly, an isothermality of < 55°C significantly and potentially favored their distribution with a mean diurnal range of 11.5 to 12.5 °C (Fig 13b).

### An. rufipes

**Fig 14:**
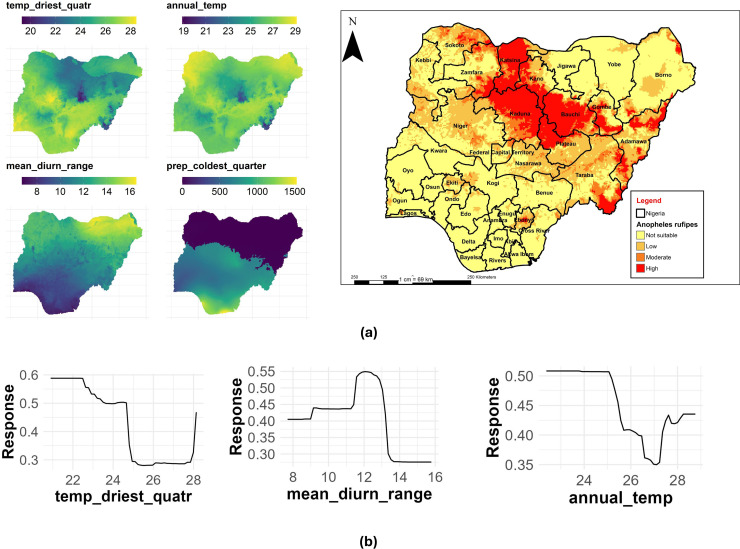
Estimates of the highest contributing variables that determines the geographical distribution of *An. rufipes.* (a) The highest environmental variables that estimate to control the geographical distribution of An. rufipes in Nigeria. Variable contributions (temperature of driest quarter, mean diurnal range and mean annual temperature), (b) response curves of three environmental predictors used in Random Forest model for *An. rufipes*. This figure was created by the authors in R programming software (R version 4.1.2, Vienna, Austria). Available at https://www.R-project.org/. The Nigerian shapefile was obtained from World Bank Data Catalog (an Open license standardized resource of boundaries (i.e., state, county) for every country in the world.

The mean temperature of the driest quarter had the highest contribution with a %IncMSE of 23.99, followed by mean diurnal range (%IncMSE of 20.34) and annual mean temperature (%IncMSE of 17.65) to the distribution of *An. rufipes* in Nigeria (Table 3). Fig 14 shows the main highest estimated environmental variables (contributions) that determine the distribution of *An. rufipes* in Nigeria. We found out that the mean temperature of the driest quarter ranged from 18.3 to 28.4 °C, mean diurnal range ranged from 7 to 16.3 °C, while annual mean temperature ranged from 18.9 to 29.1 °C, (Fig 14a). The response curves of the three variables to *An. rufipes* habitat suitability are shown in Fig 14b. These curves demonstrate that the temperature of the driest quarter ranging between 20 to 23 °C favors the potential distribution of *An. rufipes*. Similarly, a mean diurnal range of 12 to 12.4 °C significantly and potentially favored the distribution of this species with an annual mean temperature of 22 to 25 °C (Fig 14b).

### Other an non-gambiae species other than An. coustani, An. funestus, An. maculipalpis and An. rufipes

**Fig 15:**
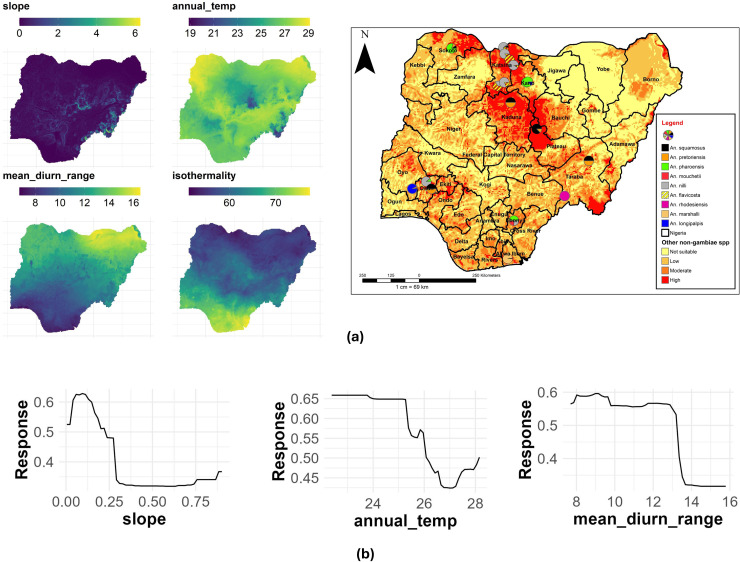
Estimates of the highest contributing variables that determines the geographical distribution of other *Anopheles* non-*gambiae* species. (a) The highest environmental variables that estimate to control the geographical distribution of other Anopheles (non-*gambiae*) species in Nigeria. Variable contributions (mean annual temperature, slope and isothermality), (b) response curves of three environmental predictors used in Random Forest model for other *Anopheles* (non-*gambiae*) species. This figure was created by the authors in R programming software (R version 4.1.2, Vienna, Austria). Available at https://www.R-project.org/. The Nigerian shapefile was obtained from World Bank Data Catalog (an Open license standardized resource of boundaries (i.e., state, county) for every country in the world.

A topographical feature (slope) had the highest contribution with a %IncMSE of 23.11, followed by annual mean temperature (%IncMSE of 22.22) and mean diurnal range (%IncMSE of 20.09) to the distribution of other *An. (*non*-gambiae)* species in Nigeria (Table 3). Fig 15 shows the main highest estimated environmental variables (contributions) that determine the distribution of other *An. (*non*-gambiae)* species in Nigeria. We found out that the slope ranged from 0(less slopy) to 6 (highly slopy), annual mean temperature ranged from 18.9 to 29.1 °C, while mean diurnal range ranged from 7 to 16.3 °C, (Fig 15a). The response curves of the three variables to other *An. (*non*-gambiae)* species habitat suitability are shown in Fig 15b. These curves demonstrate that less slopy areas favors the potential distribution of these *An. (*non*-gambiae)* species. Similarly, an annual mean temperature of 23 to 25 °C, significantly favored their distribution with a mean diurnal range of 8 to 13 °C (Fig 15b).

## Discussion

In Nigeria, significant progress has been made towards generating quality data for decision making. This progress can be attributed to deployment of vector control tools alongside effective case management. The importance of vector surveillance in planning, implementation, monitoring and evaluation of vector control interventions cannot be overemphasized [10]. Hence, gaining a comprehensive insight into the distribution patterns of all malaria vector populations across an entire nation is a crucial asset in the fight against malaria transmission. We have previously reported the occurrence data for 3 major malaria vectors in Nigeria [10], and our current study presents the occurrence data for other malaria vectors from surveillance studies in 20 out of 36 states in Nigeria. We also predicted distribution and relative abundance for the entire country using the random forest modeling approach.

To the best of our knowledge, this is the first study to report the occurrence and model-based estimates of these important secondary malaria vectors for the entire country, employing contemporary and very recent data along with similar methods of data collection. About 13 different non-*gambiae* species of *Anopheles* were encountered in this study, with most of them coexisting in sympatry at various locations. These mosquitoes were found in 10 out of the 20 states, spanning different ecological zones in the country. From the earlier study, *An. gambiae* was found in all study locations, including states where non-*gambiae* species were not found. The detailed distribution of *An. gambiae* has been published elsewhere [10].

The widespread presence of non-*gambiae* species across many parts of the country emphasizes the need for increased attention to these species at the national level, as they may play a role in malaria transmission, contributing to the persistent prevalence of malaria in Nigeria despite vector control efforts. Previous localized studies have implicated these secondary vectors in malaria transmission [13–15]. Also, studies from other African countries have suggested that these species could exhibit higher vectorial capacity than the dominant vectors (*An. gambiae*) and even maintain high malaria transmission during the dry season [31,32]. Hence, understanding their distribution and spread in the country will help shape the focus of vector control in the country.

The fact that the non-*gambiae* species of *Anopheles* exhibit more occurrence in the Northern parts of the country, albeit with lower numbers, while they appear to be confined to fewer locations in the southern parts, but with higher numbers suggests that these mosquitoes possess high adaptive abilities, demonstrating elevated relative population densities in areas where suitable habitats are limited. Nevertheless, it has been suggested that even at far lower densities, some of these species still have a very high tendency to transmit malaria [31,33,34] especially during the dry season. One of the prominent vectors identified with high density in this study is *An. funestus* and *An. coustani*. These mosquitoes have a wide distribution range in the northern parts of the country, while being confined to limited areas in the south but with higher density. *An. coustani* and *An. funestus* are known to be efficient vectors of *Plasmodium*, transmitting outdoor and indoor respectively [31,35]. Their wide distribution in the northern part of the country might also be due to large-scale rice farming, which provides suitable breeding sites for mosquitoes throughout the year. For example, *An. funestus* and *An. coustani* are known to breed in water collections covered with vegetation around rice farms and streams [31,33].

Apart from *An. coustani* and *An. funestus*, other vectors encountered in this study have been implicated in malaria transmission in other countries. For instance, *An. rufipes* has been reported in Zambia and Kenya [36,37], and *An. maculipalpis* in Kenya [37]. Furthermore, in the forested areas of Cameroon, *An. moucheti* was reported as the predominant and most prevalent malaria vector, while *An. marshalli*, *An. nilli*, *An. pretoriensis*, and *An. squamosus* were considered secondary malaria vectors in Cameroon, Kenya, and Ethiopia [38]. This highlights the importance of active surveillance and the production of risk maps to precisely identify focus areas for targeted interventions.

The major focus in vector surveillance and control in Nigeria has been on members of the *An. gambiae* complex [10]. However, our current studies indicate the need to also consider and increase surveillance efforts targeting these other vectors. Currently, little data are available about their involvement in malaria transmission, especially in the dry season across the country. Moreover, these species are known to be more abundant during the dry season, with peak density occurring during the transition from the dry season to the rainy season due to their larval habitat stability and adaptation to desiccation [39,40]. This calls for more deliberate surveillance if we are going to win the war against malaria in the country.

Geospatial modeling is one approach that can be used to enhance vector and disease surveillance in low- to middle-income countries. It provides valuable information on the possible spread of disease vectors, thus informing how the already limited control resources can be maximally utilized within the country [17]. In this study, our model suggested a wide-ranging distribution of these non-*gambiae* species of *Anopheles* in the country, covering 33 out of the 36 states. This finding emphasizes the need for increased surveillance and further studies on the transmission dynamics of these species as suitability data alone does not suffice for transmission. This information is crucial for designing tailored control tools, as these species exhibit different behaviors (e.g., outdoor biting [35]) compared to the current control strategies in the country, which mainly target indoor-biting mosquitoes (i.e., LLIN and IRS). We were able to produce a country wide model-based distribution for four distinct species (*An. coustani, An. funestus, An. rufipes* and *An. maculipalpis*) based on availability of occurrence points. Of the four species, *An. coustani* seems to have the most restricted distribution confined to large part of six states mainly located in the northern parts of the country. However, some fewer parts of other states including the southern parts of the country were also predicted to be suitable for this *An. coustani.* This emphasizes the need for increased vigilance and studies, as *An. coustani* has been implicated as a potential malaria vector in Nigeria [13–15] and other African countries [41–44].

The potential for higher range expansion demonstrated by *An. funestus, An. maculipalpis, and An. rufipes* should serve as a warning to pay close attention to these mosquitoes and their role in malaria transmission. *An. funestus*, in particular, has been acknowledged as one of the primary species in sub-Saharan Africa [32] and in Benin Republic, which shares a border with Nigeria [41]. They exhibit high anthropophilic tendencies and contribute significantly to malaria transmission, especially during the dry season, where they might even outcompete the supposed ‘dominant’ malaria vector, *An. gambiae* s.l. [45]. Thus, the potential spread of *An. funestus* and other species across the country might also explain the persistent burden of malaria in some parts of the country. Therefore, more efforts should be tailored towards controlling these mosquitoes, which often bite outdoors rather than indoors. One effective method of control is larval source management, which does not discriminate between outdoor and indoor biting mosquitoes.

Overall, the non-*gambiae* species were predicted to exhibit a high population spread across the country. The model has assisted us in pinpointing hotspots for these species at the state level, enabling informed and cost-effective decisions regarding the allocation of control resources. For example, while nearly the entire Oyo State is anticipated to have a high relative proportion of *An*. *funestus*, specific areas in Ogun, Taraba, and Kebbi states were projected to potentially harbor a large population of *An. funestus*. This information guides tailored control interventions with the limited available resources.

It is quite noteworthy to state that the distribution of the four species (*An. coustani, An. funestus, An. rufipes* and *An. maculipalpis*) were majorly influenced by temperature rather than precipitation related factors. These factors include mean temperature of the driest quarter of the year, isothermality (a measure of day-to-night temperature variations within a month relative to the year), annual mean temperature and mean diurnal range (a measure of relative temperature fluctuations in a month). The range of these factors seems to be quite similar across all the species. This finding is in contrast with our previous work on predictive modeling of *An. gambiae* in Nigeria which indicated both rainfall and temperature related factors as major contributing environmental variables influencing their distribution [10]. This is not totally surprising as studies have shown that these secondary malaria vectors can even thrive well even in dry season because of their breeding preferences. For instance, in Benin, *An. funestus* was only reported in districts characterized by a long dry season and a very short rainy season. This can also partly explain why they have more occurrence locations in the Northern part of the country [32,45].

This result underscore the importance of understanding how environmental factors, particularly climate and topography may play a role when designing and implementing malaria control strategies in Nigeria. Analyzing the distribution patterns and environmental preferences of mosquito species is crucial for strategically deploying interventions like insecticide-treated bed nets, indoor residual spraying, and larval source management. This approach, particularly targeted at the most vulnerable areas, enhances the effectiveness of malaria control efforts [10]. For example, targeting *An. funestus* through larviciding or aerial spraying during periods of low temperatures in the driest quarter, coupled with areas exhibiting lower temperature fluctuations (isothermality), could yield significant impact. This targeted approach, especially in identified hotspots, allows for the efficient utilization of limited resources while maximizing the benefits. It further emphasizes that enhancing the efficacy of malaria control initiatives in the country should involve continuous monitoring and adjustment of control strategies in response to variations in environmental conditions and vector behavior.

This work is not without limitations; one significant limitation is the exclusion of physico-chemical parameters of larval habitats, such as pH, dissolved oxygen, total dissolved solids, electrical conductivity, turbidity, and demographic variables like the presence of cattle, farming activities, and human population from the model. These variables are also important and may offer explanations for the distribution of these mosquitoes. Therefore, there is a need to incorporate these variables and evaluate their effects on the distribution of non-*gambiae* species of *Anopheles* in our future research. The second limitation is the limited occurrence points for these species due to surveillance shortages. Although the modeling approach can handle this relatively small sample size (evident in the good model performance metrics), there is a possibility that more areas might be suitable for these species once we input more data into the model. Therefore, there is a need to systematically survey more areas in the country to address any shortfall from the currently available data.

For the first time, we have successfully predicted the potential distribution and relative density of non-*gambiae* species of *Anopheles* across Nigeria, utilizing real-time data collected through standardized and uniform mosquito collection methods across the country.

## Conclusion

Our study presents comprehensive data on secondary malaria vectors in Nigeria, with *An. coustani, An. funestus, An. rufipes,* and *An. maculipalpis* identified as the most common species found across the country in sympatry. The model indicates a wide-ranging distribution of these non-*gambiae Anopheles* species, covering 33 out of the 36 states. Particularly, the species-specific model highlights the extensive range expansion of *An. funestus* compared to others. Temperature, rather than rainfall, emerges as the primary environmental variables influencing their distribution. Emphasizing the importance of active surveillance in vector control, we have established a model-based baseline for the species distribution of non-*gambiae* malaria vectors in the country, derived from empirical data. Recognizing the time and cost challenges of country-wide vector mapping, these additional maps can help shape the country program in resource allocation for effective vector control. To better understand the ecology of these secondary vectors, additional research is needed to examine the influence of variables such as land use or land cover, vegetation index, livestock density and the coverage of vector control interventions on their distribution.

## Supporting information

S1 File Study protocol for CDC light trap, pyrethrum spray catch and larval collection. (PDF)

S2 File Total number of Non-gambiae species caught for each year across the study locations. (PDF)

S3 File Total number of Non-gambiae species caught by species and trapping methods. (PDF)

S4 File Monthly catches of Non-gambiae species for CDC LT and PSC methods of collection for Year 2020, 2021 and 2022 (Figs 1–30). (PDF)
