# Supplementary material for: Spatial distribution and geospatial modeling of potential spread of secondary malaria vectors species in Nigeria using recently collected empirical data
Source: PLoS One. 2025 Apr 21;20(4):e0320531. doi: 10.1371/journal.pone.0320531 (PMC12011306; doi:10.1371/journal.pone.0320531)
Supplement: S1 File — (PDF) [file pone.0320531.s001.pdf]

Spatial Distribution and Geospatial Modeling of Potential Spread of Secondary Malaria Vectors Species in Nigeria using recently collected empirical data

Study protocol for CDC light trap, pyrethrum spray catch and larval collection

S1

Methods of Adult Mosquito Collection

Two methods of adult mosquito collection—CDC Light Trap (CDC LT) and Pyrethrum Spray Catch (PSC)—were employed in this study to target outdoor and indoor mosquitoes, respectively. The methods followed that of Yohannes and Boelee (2011). This same method was also used by Saili *et al.,* (2023). The procedure was carried out every month for 3 years (2020-2022). The details are highlighted below;

CDC Light Trap (CDC LT)

The CDC light traps were set up to collect both indoor and outdoor mosquitoes. Each trap was baited using a human volunteer who slept near the trap to attract mosquitoes. The volunteer did not use a treated bed net (but slept under untreated net, with the CDC LT hung close to the net) to avoid interference with mosquito behavior and trapping efficiency. The traps were placed at four houses per sentinel site (State) and operated for three nights each month. On each night, eight traps were deployed, two per house (One indoor and One Outdoor). Collection cups were checked and replaced hourly throughout the night, typically from 7:00 PM to 6:00 AM.

Pyrethrum Spray Catch (PSC)

The PSC method was used to collect indoor resting mosquitoes. Sampling was conducted by selecting thirty-two houses per sentinel site (State), ensuring that these houses were different from those used for CDC light traps. The sample collection was done over a period of 3 days (i.e. 10-11 houses per day). Residents were asked to vacate the house early in the morning (around 6:00 am) before spraying began. White sheets were laid on the floor and furniture to collect fallen mosquitoes. Pyrethrum insecticide was sprayed in the rooms, and the sheets were examined after 10–15 minutes to collect the mosquitoes. This process was repeated monthly.

Sampling Design and Standardization

- **Trap Placement and Frequency**: A total of 24 CDC light trap collections were performed each month in each sentinel site (8 traps × 3 nights), while PSC sampling involved 32 houses per site each month.
- **Volunteer Conditions**: The volunteer bait for the CDC light traps slept in close proximity to the traps, without a insecticide treated bed net, under a standardized protocol to ensure consistency in mosquito attraction across sentinel sites.

- **Larval Sampling** : For larval sampling, breeding sites were first identified through local knowledge and larval prospection. Sampling was done proportionally to the size of the breeding site, ensuring larger sites were adequately represented. Standard dippers were used to collect larvae, with three dips per square meter taken from the edges and middle of each site to ensure representative sampling.
- **Number of people in a team**: Each team comprises of 15 members, this is to ensure proper and standardized data collection and quality assurance.

References:

1. YOHANNES, M., & BOELEE, E. (2011). Early biting rhythm in the afro-tropical vector of malaria, *Anopheles arabiensis,* and challenges for its control in Ethiopia. Medical and Veterinary Entomology, 26(1), 103–105. doi:10.1111/j.1365-2915.2011.00955.x
2. Saili, K., de Jager, C., Sangoro, O.P. et al. Anopheles rufipes implicated in malaria transmission both indoors and outdoors alongside Anopheles funestus and Anopheles arabiensis in rural south-east Zambia. Malar J 22, 95 (2023). https://doi.org/10.1186/s12936-023-04489-3
