# Supplementary material for: Spatial distribution and geospatial modeling of potential spread of secondary malaria vectors species in Nigeria using recently collected empirical data
Source: PLoS One. 2025 Apr 21;20(4):e0320531. doi: 10.1371/journal.pone.0320531 (PMC12011306; doi:10.1371/journal.pone.0320531)
Supplement: S2 File — (PDF) [file pone.0320531.s002.pdf]

S2: Total number of Non-gambiae species caught for each year across the study locations

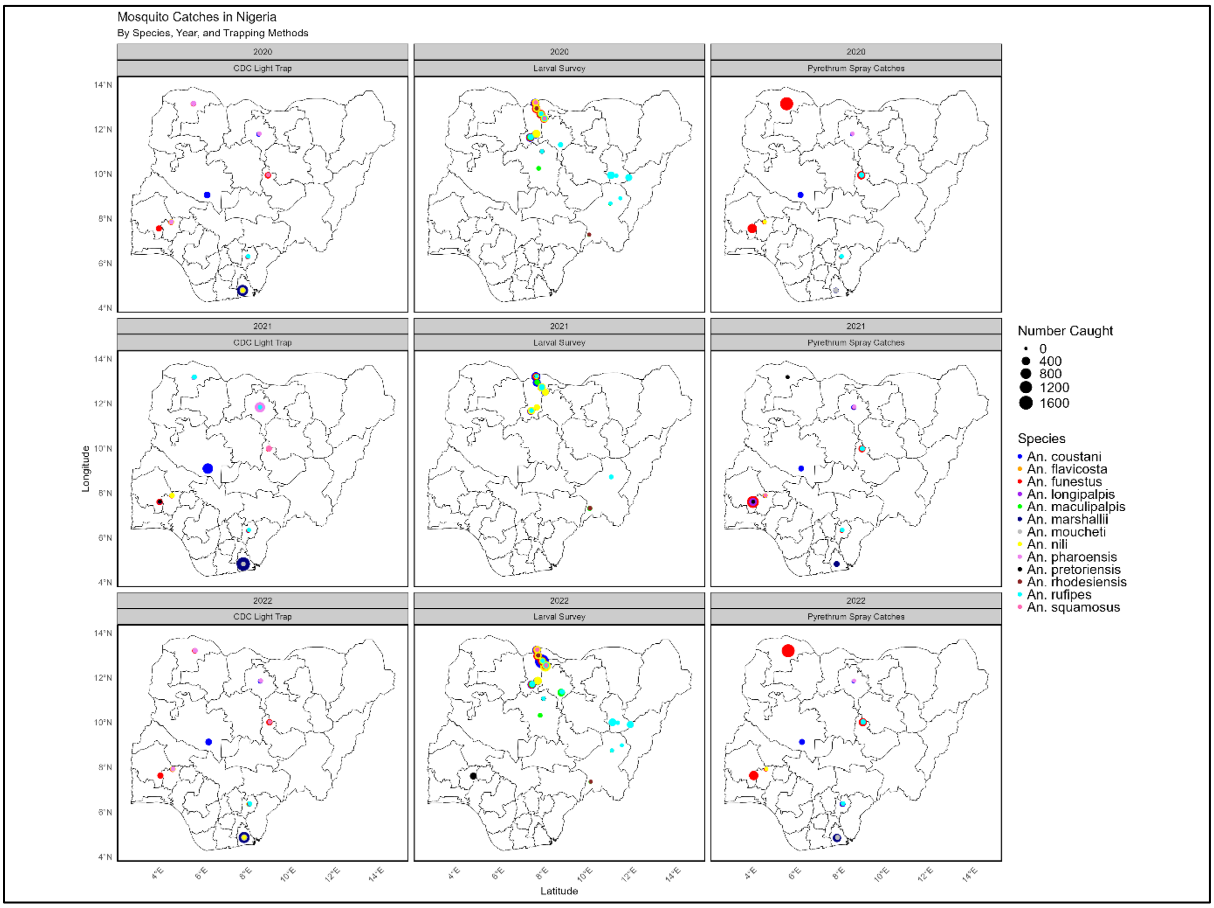

This figure was created by the authors in R programming software (R version 4.1.2, Vienna, Austria). Available at https://www.R-project.org/. The Nigerian shapefile was obtained from World Bank Data Catalog (an Open license standardized resource of boundaries (i.e., state, county) for every country in the world).
