# Supplementary material for: Spatial distribution and geospatial modeling of potential spread of secondary malaria vectors species in Nigeria using recently collected empirical data
Source: PLoS One. 2025 Apr 21;20(4):e0320531. doi: 10.1371/journal.pone.0320531 (PMC12011306; doi:10.1371/journal.pone.0320531)
Supplement: S4 File — (PDF) [file pone.0320531.s004.pdf]

S4: Monthly catches of *Non-gambiae* species for CDC LT and PSC methods of collection for Year 2020, 2021 and 2022 (Figures 1 – 30)

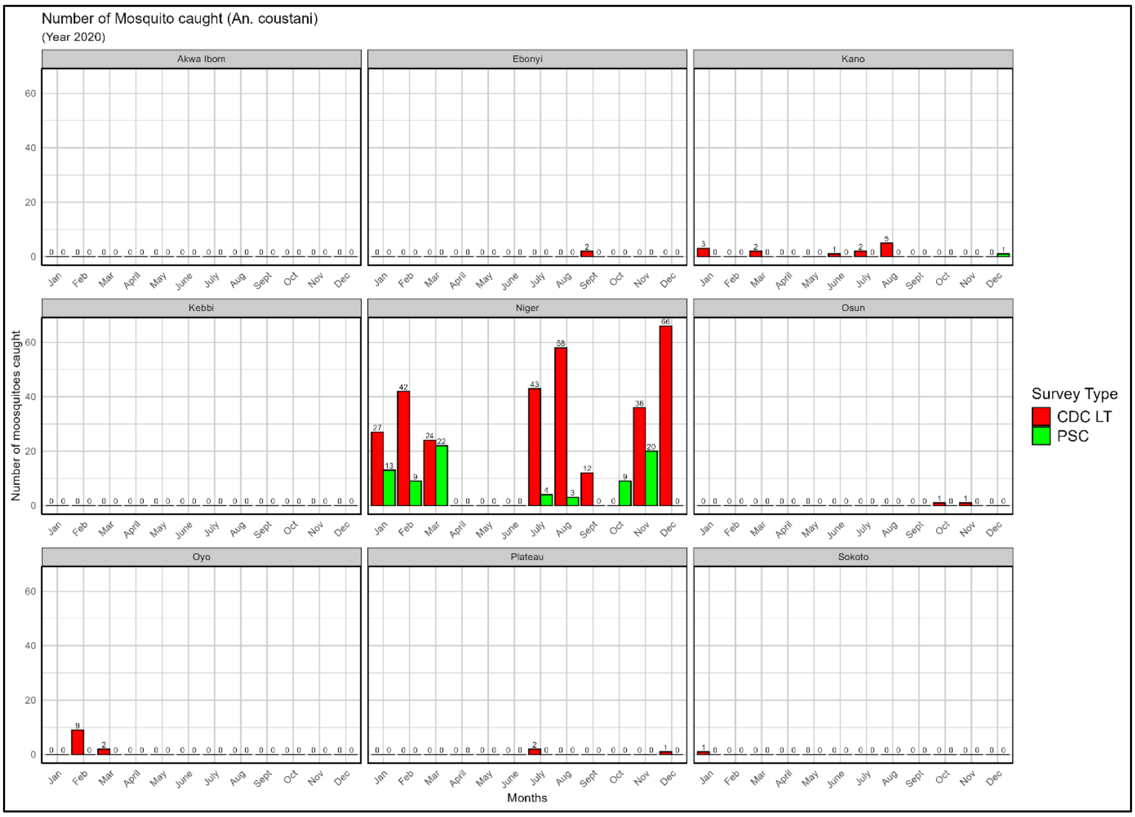

Figure 1: Monthly catches of *An. coustani* for CDC LT and PSC methods of collection for Year 2020

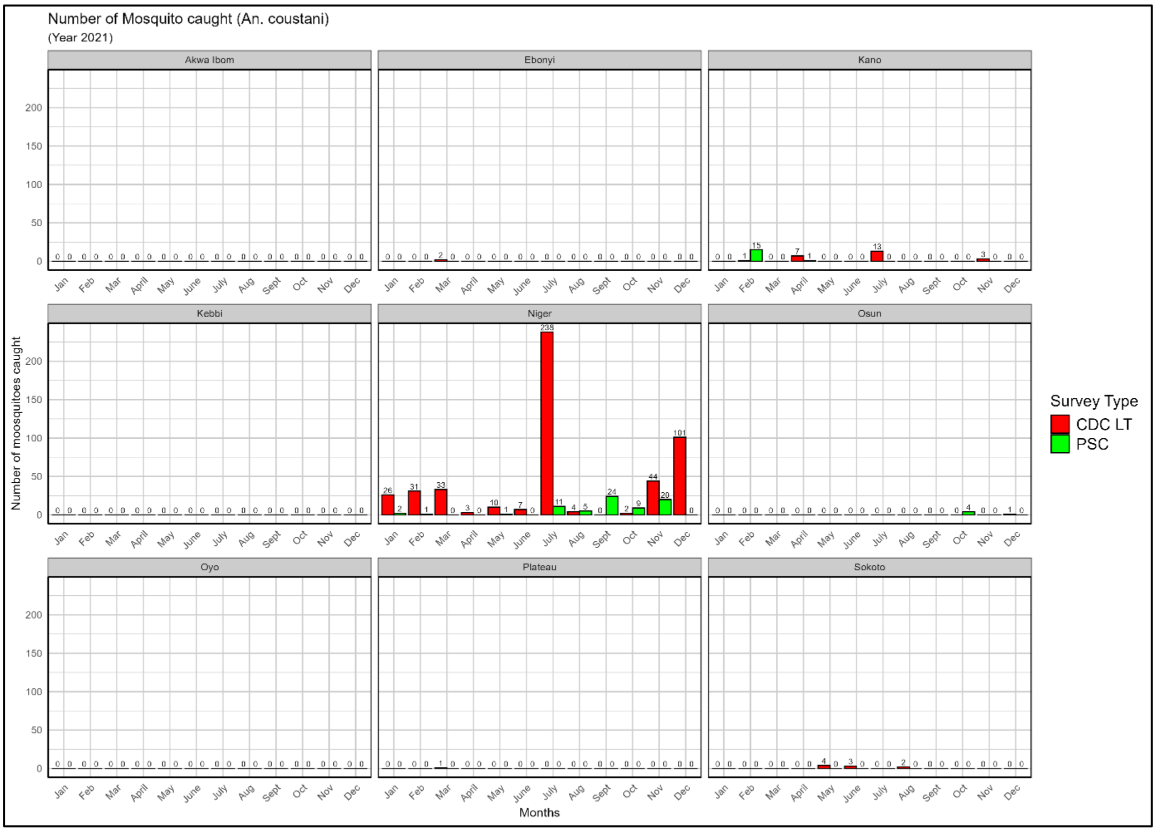

Figure 2: Monthly catches of *An. coustani* for CDC LT and PSC methods of collection for Year 2021

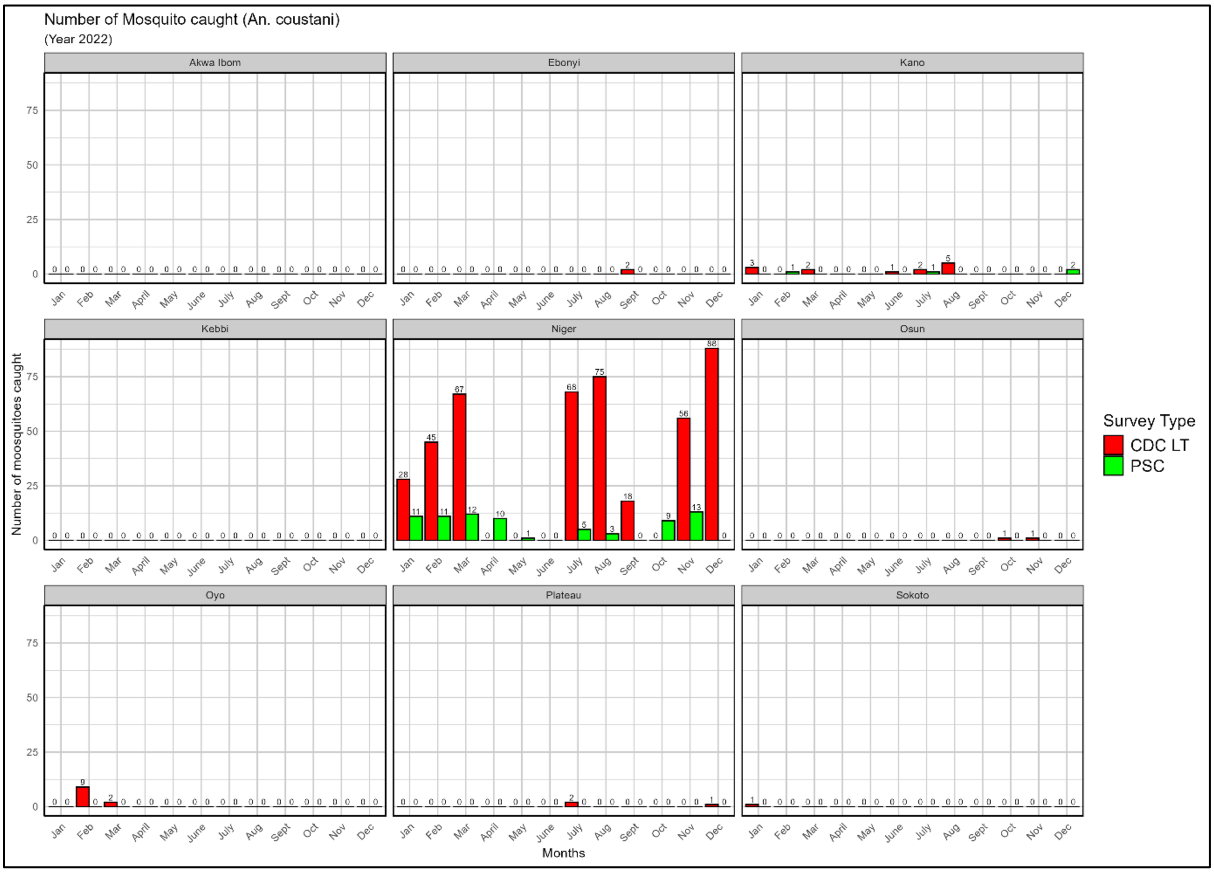

Figure 3: Monthly catches of *An. coustani* for CDC LT and PSC methods of collection for Year 2022

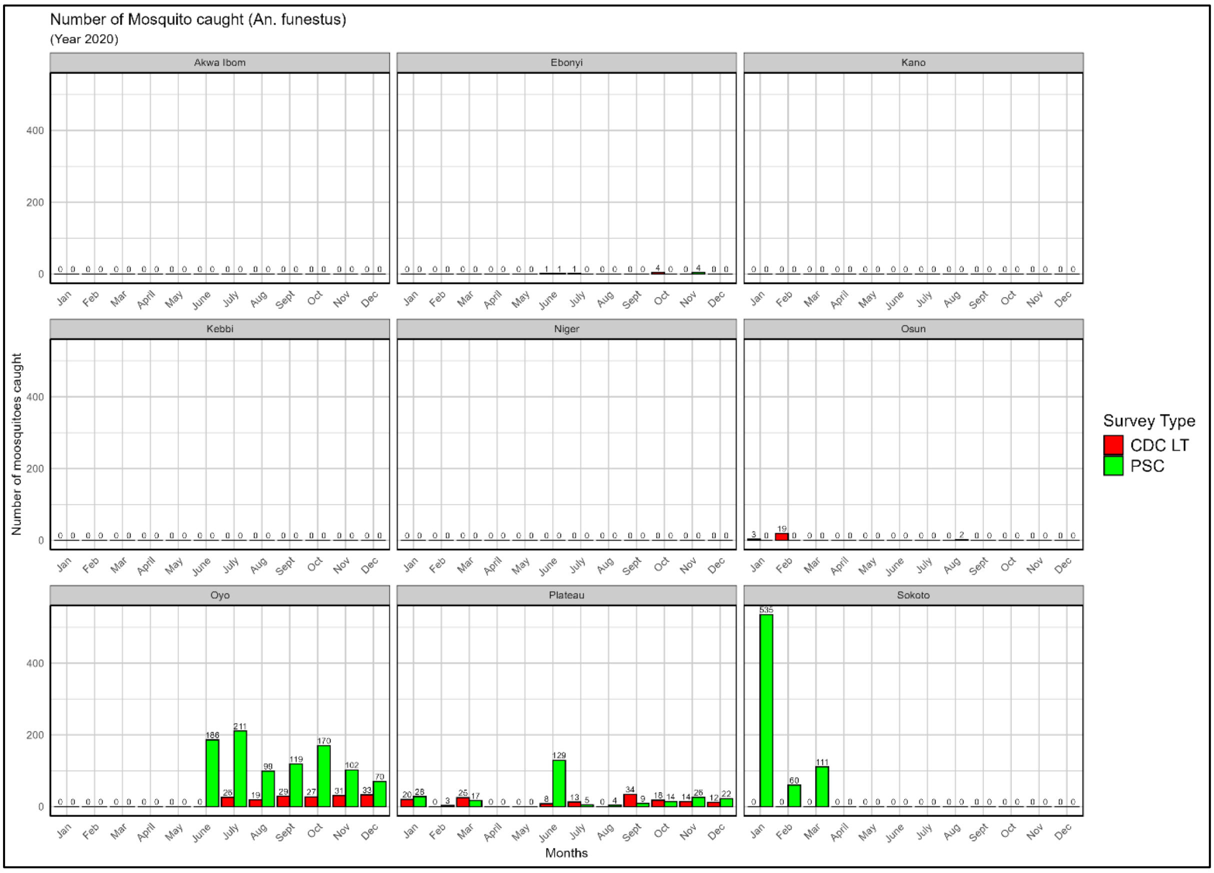

Figure 4: Monthly catches of *An. funestus* for CDC LT and PSC methods of collection for Year 2020

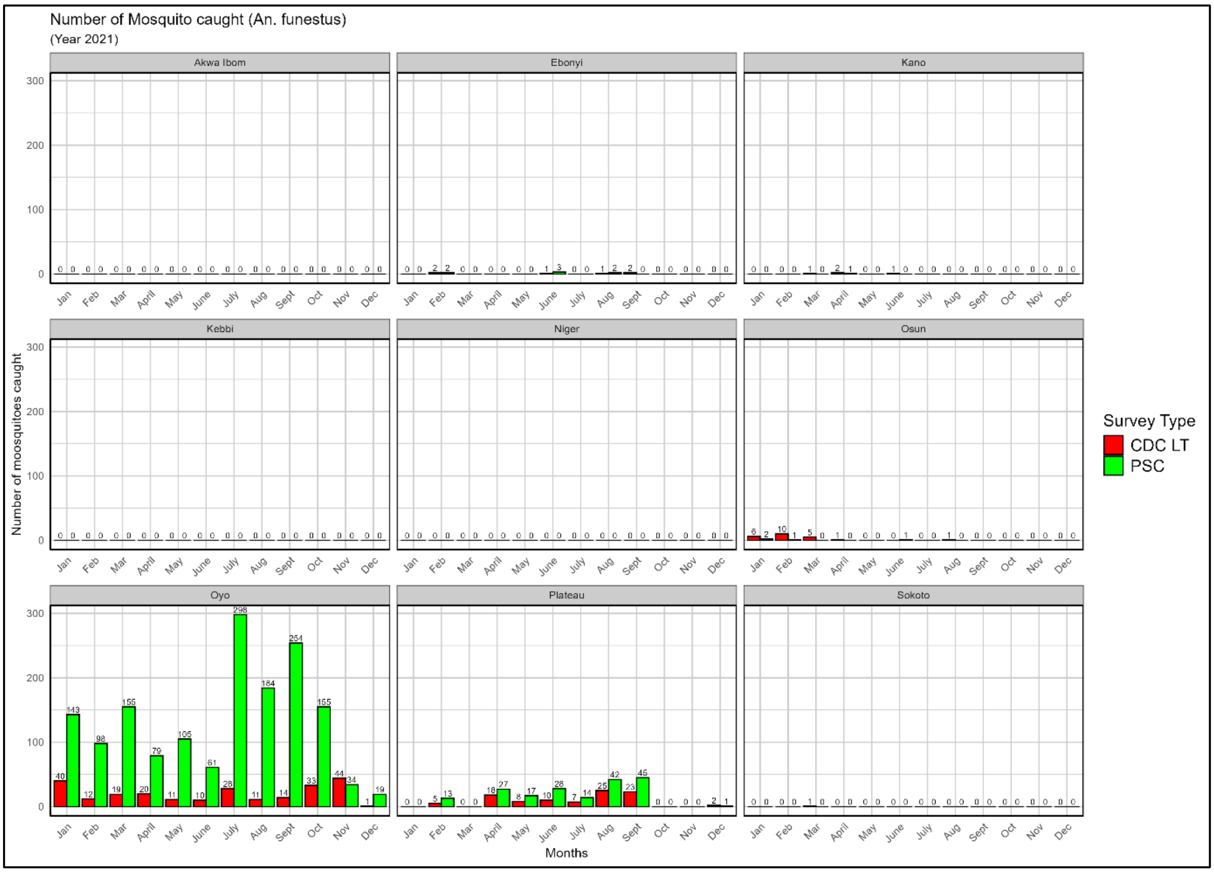

Figure 5: Monthly catches of *An. funestus* for CDC LT and PSC methods of collection for Year 2021

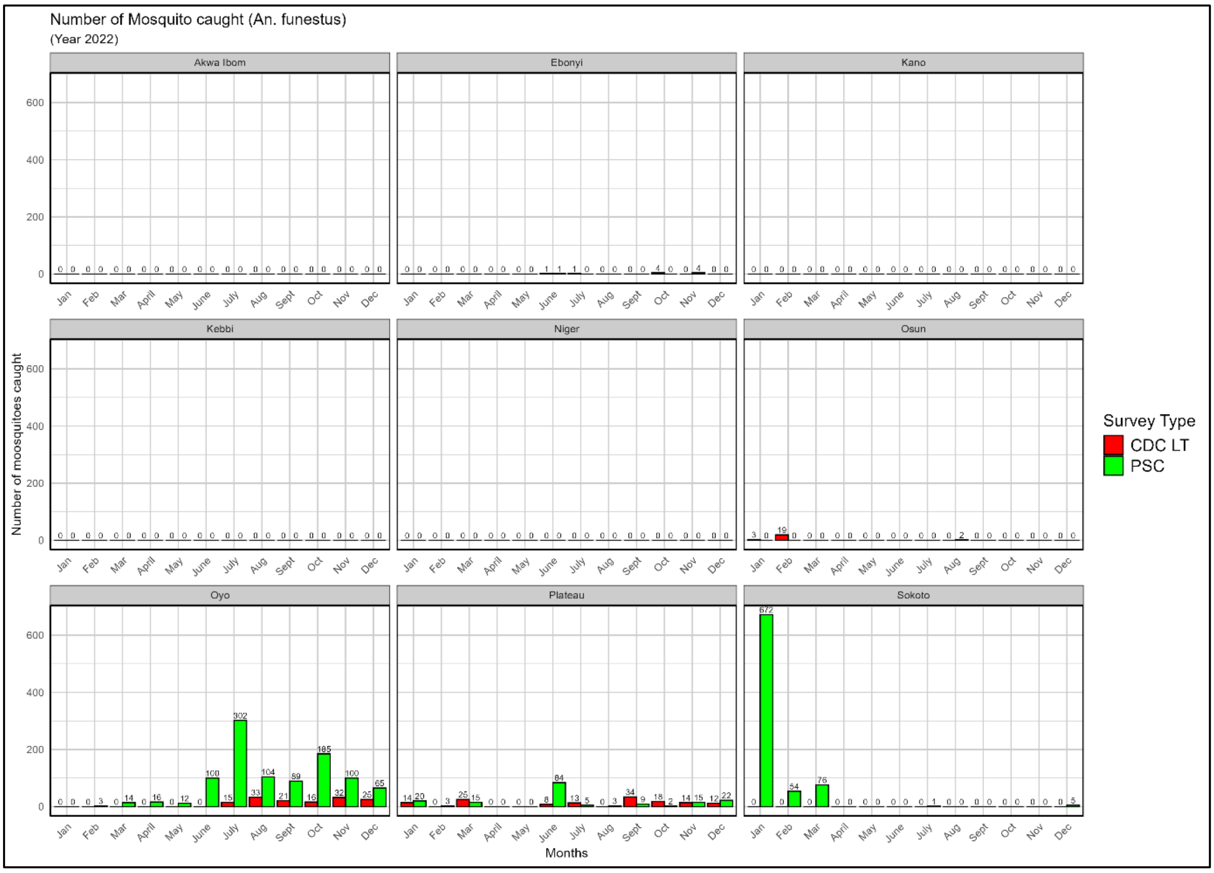

Figure 6: Monthly catches of *An. funestus* for CDC LT and PSC methods of collection for Year 2022

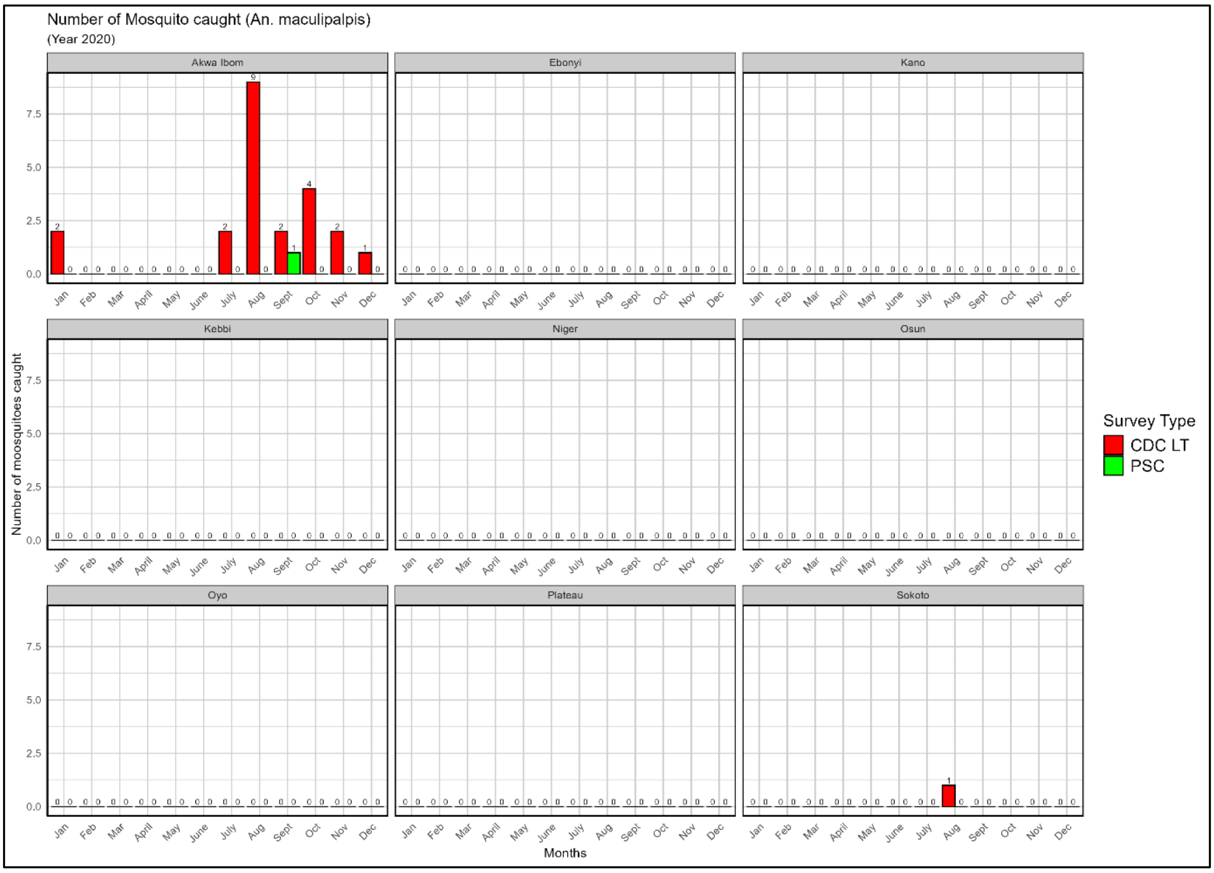

Figure 7: Monthly catches of *An. maculipalpis* for CDC LT and PSC methods of collection for Year 2020

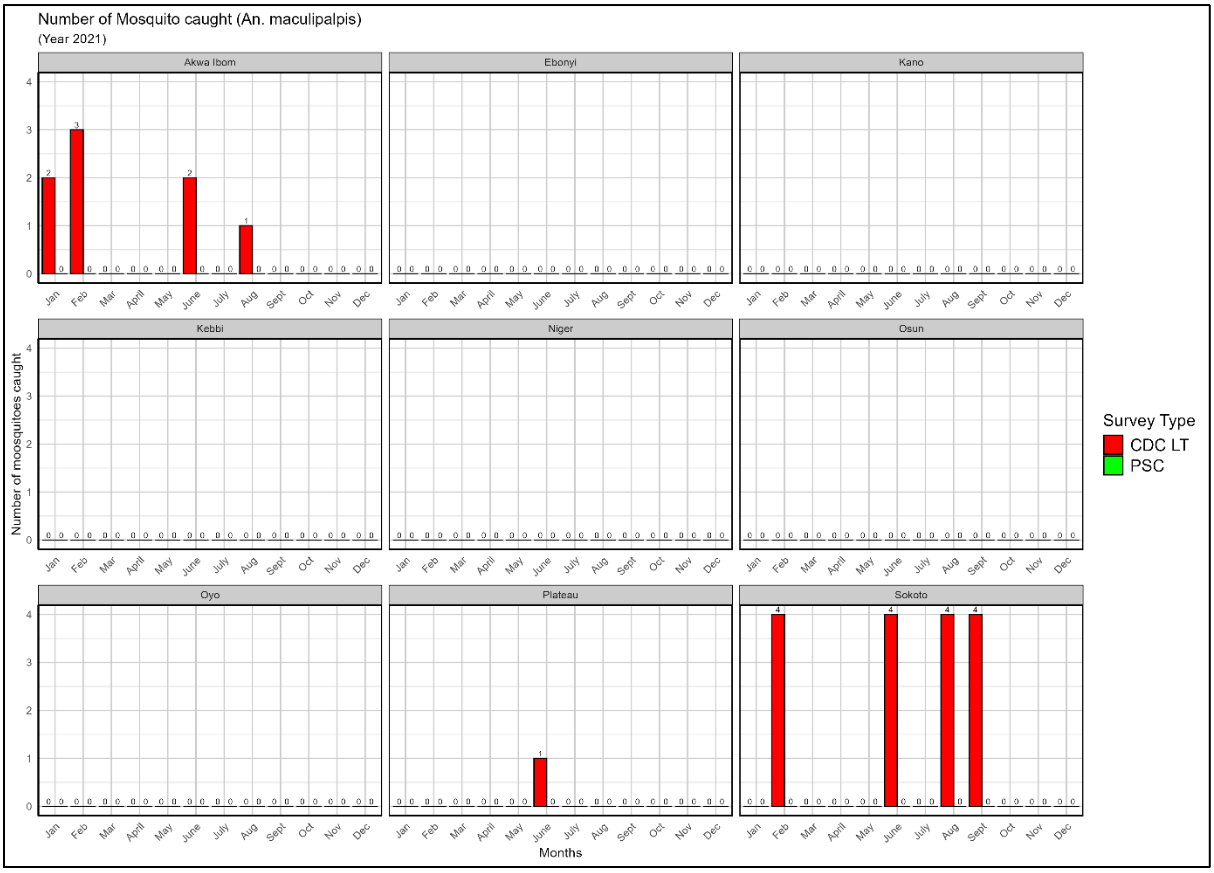

Figure 8: Monthly catches of *An. maculipalpis* for CDC LT and PSC methods of collection for Year 2021

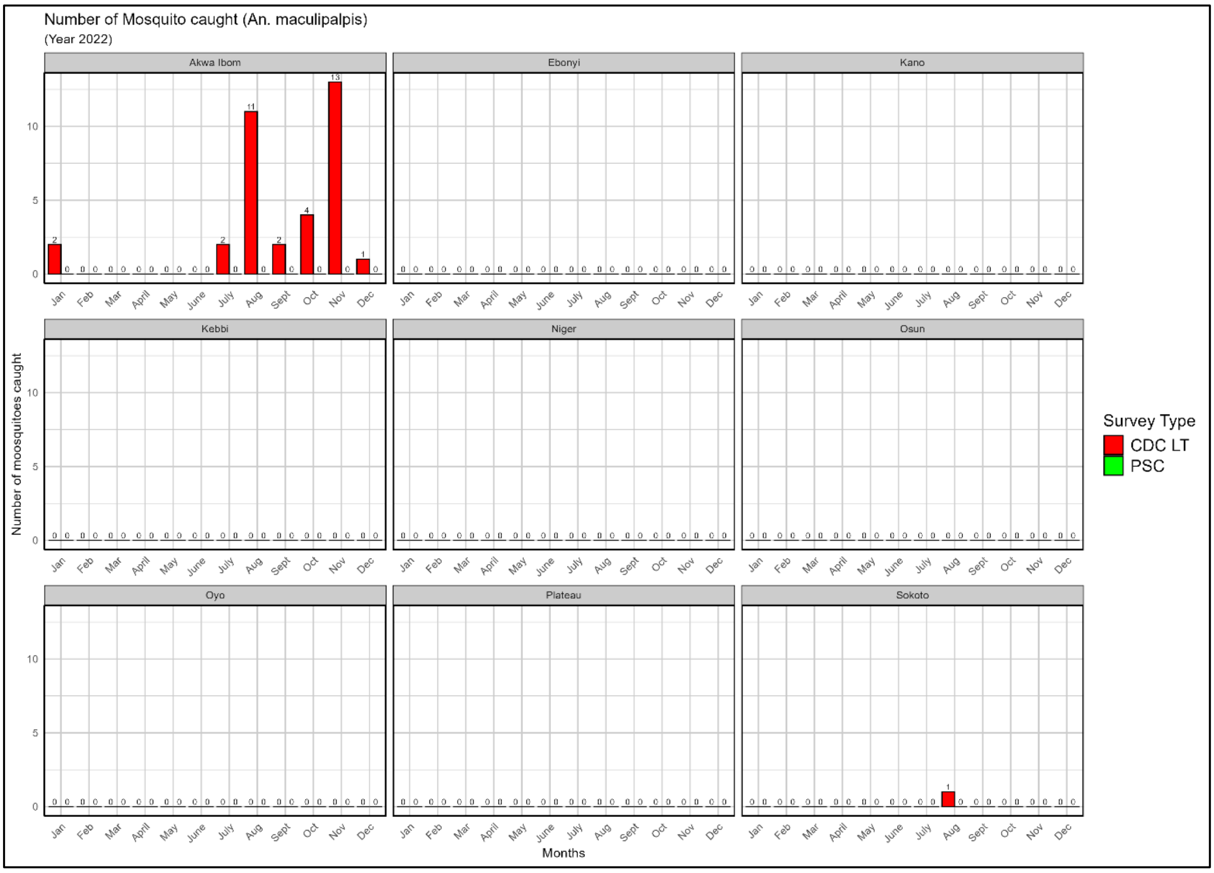

Figure 9: Monthly catches of *An. maculipalpis* for CDC LT and PSC methods of collection for Year 2022

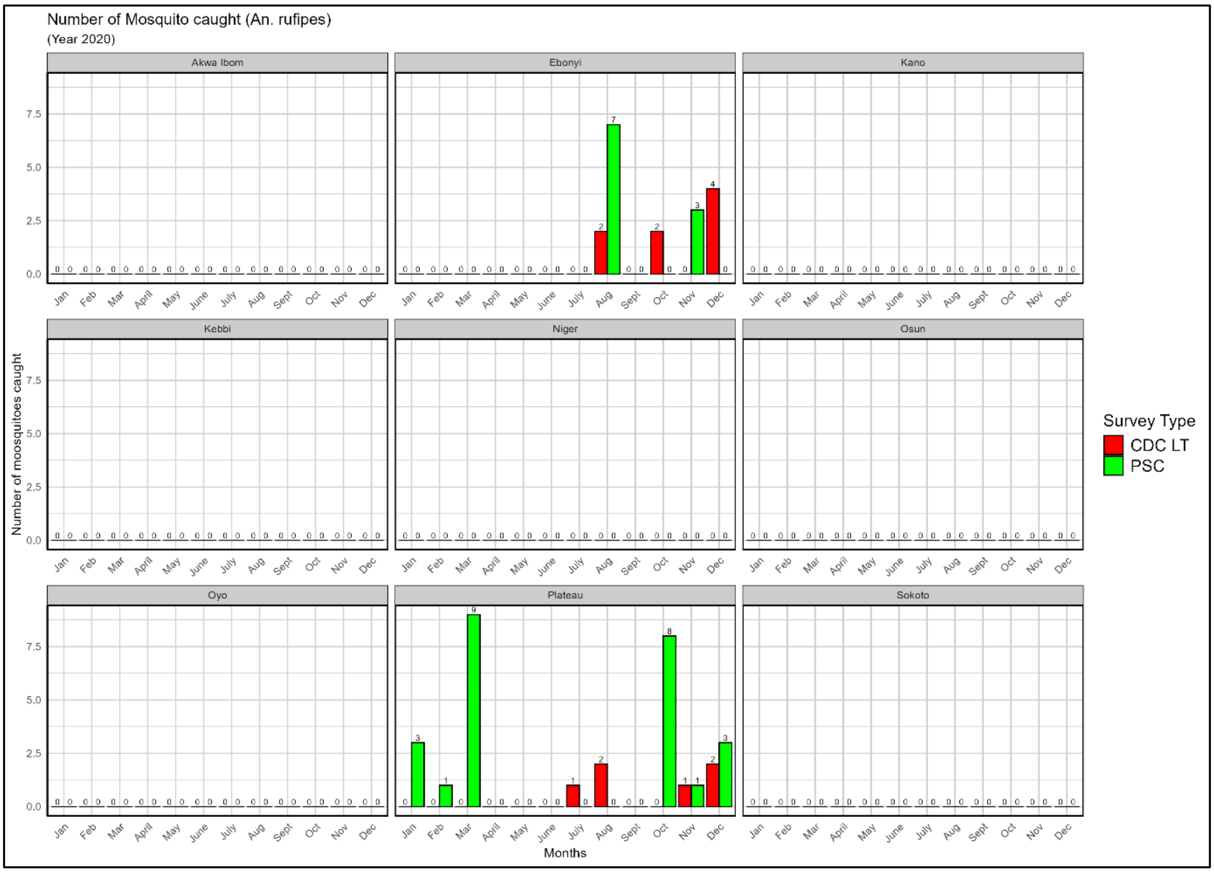

Figure 10: Monthly catches of *An. rufipes* for CDC LT and PSC methods of collection for Year 2020

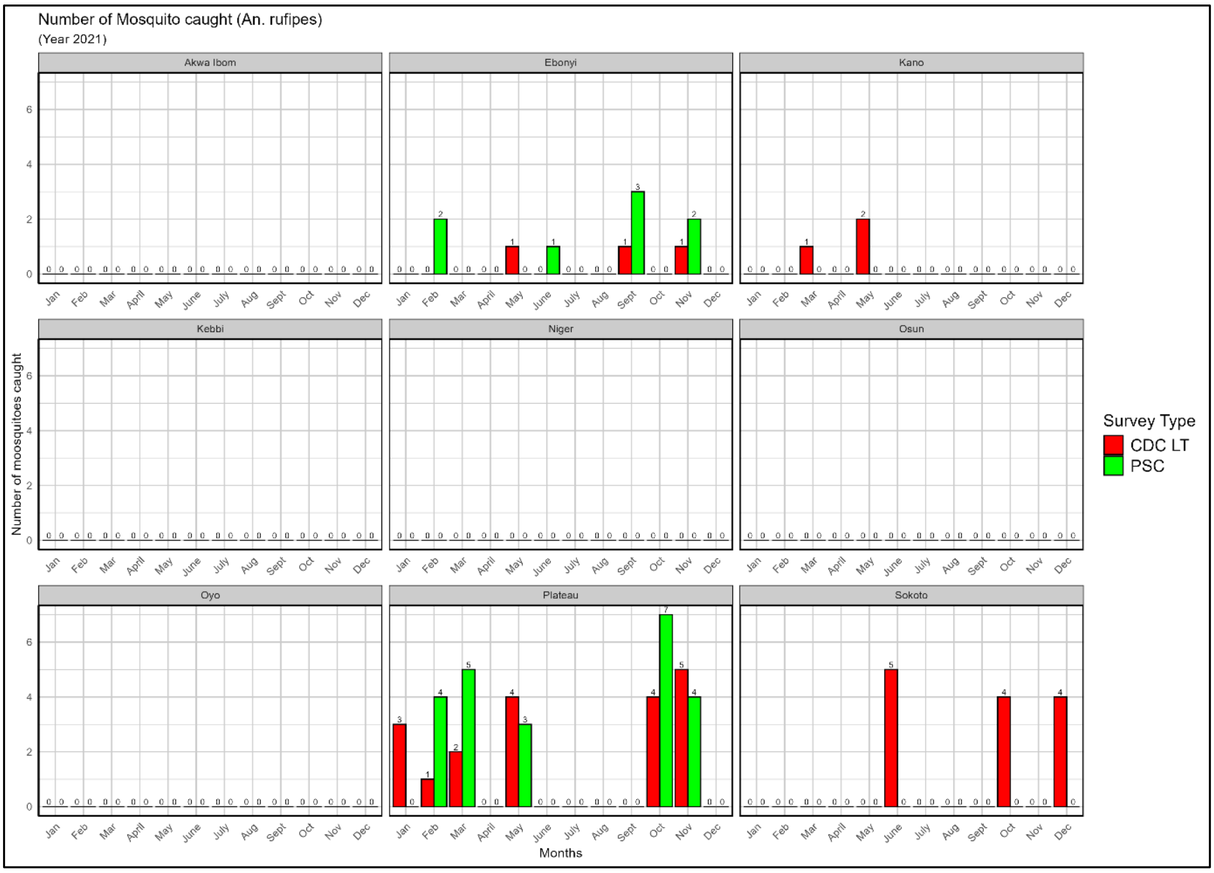

Figure 11: Monthly catches of *An. rufipes* for CDC LT and PSC methods of collection for Year 2021

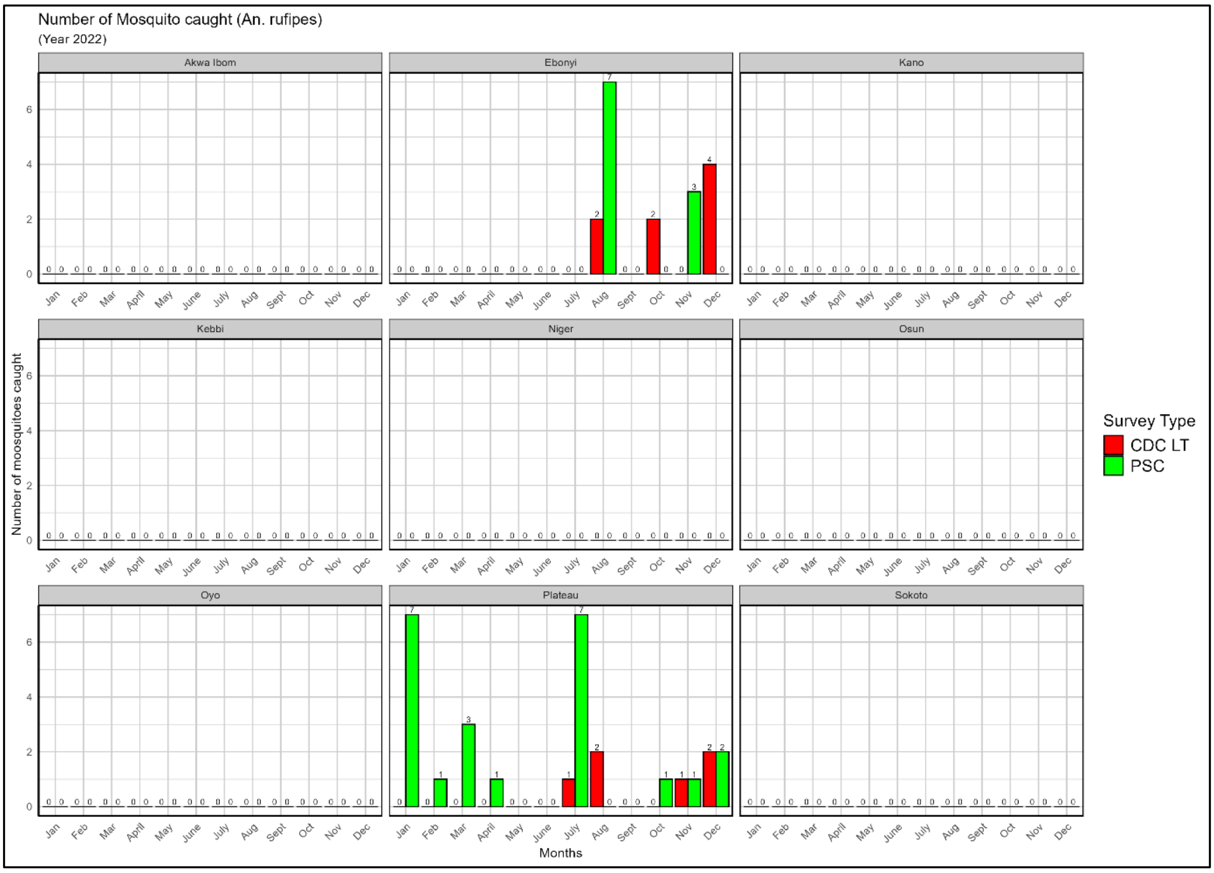

Figure 12: Monthly catches of *An. rufipes* for CDC LT and PSC methods of collection for Year 2022

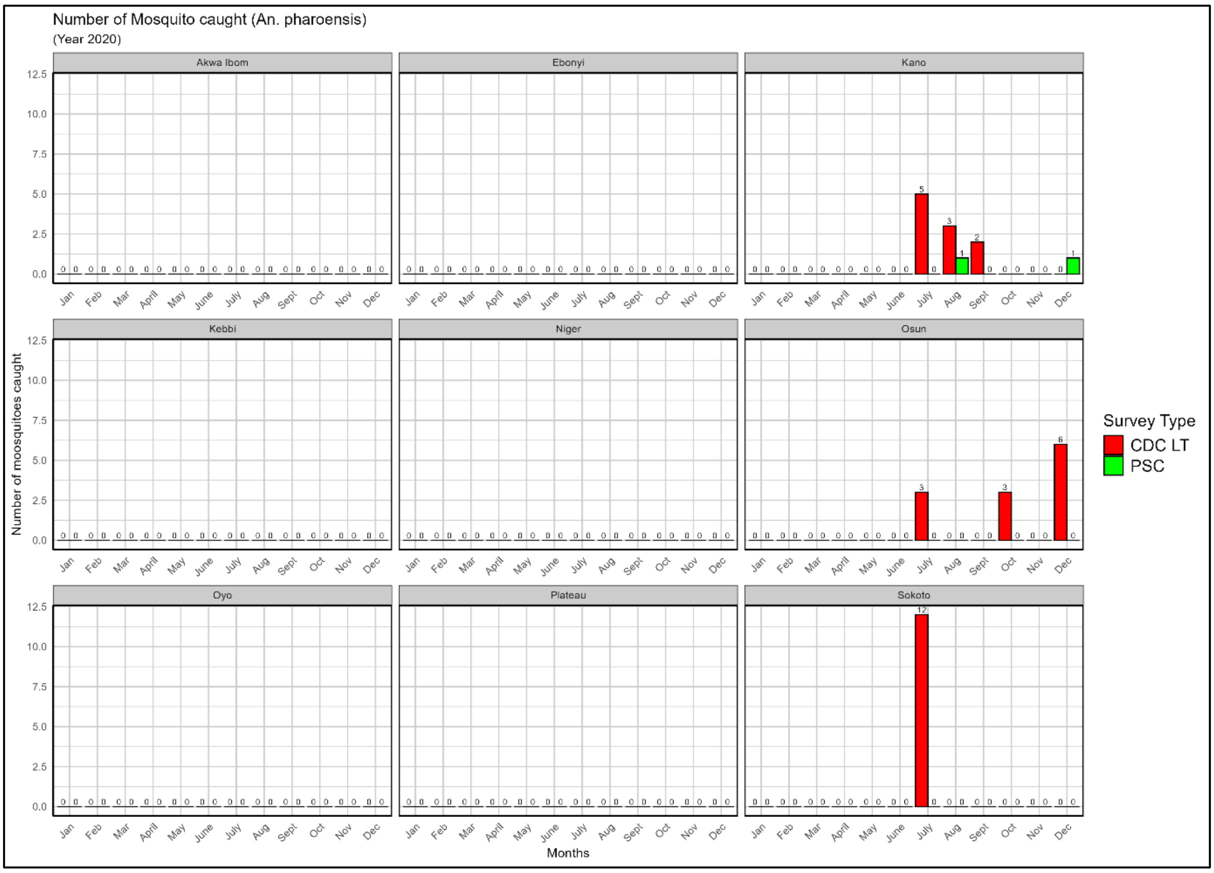

Figure 13: Monthly catches of *An. pharoensis* for CDC LT and PSC methods of collection for Year 2020

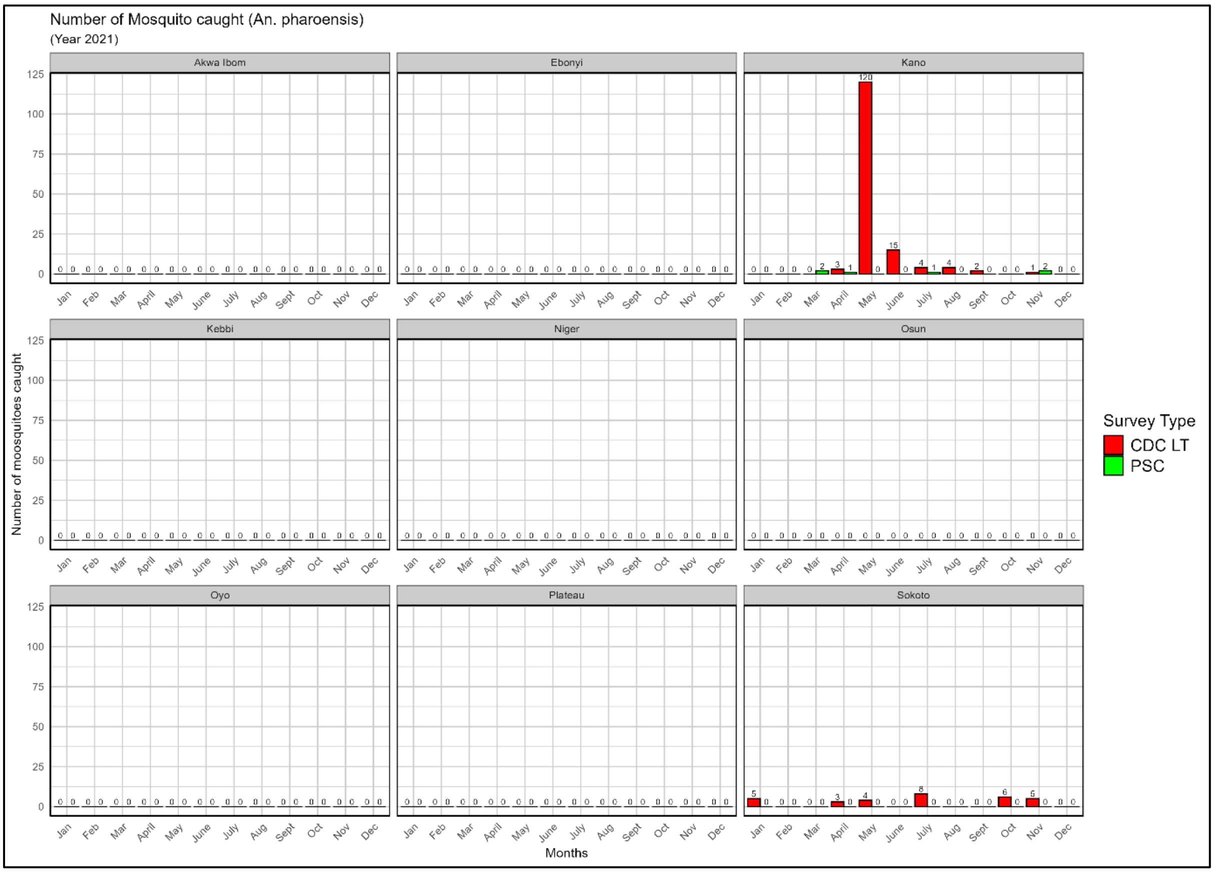

Figure 14: Monthly catches of *An. pharoensis* for CDC LT and PSC methods of collection for Year 2021

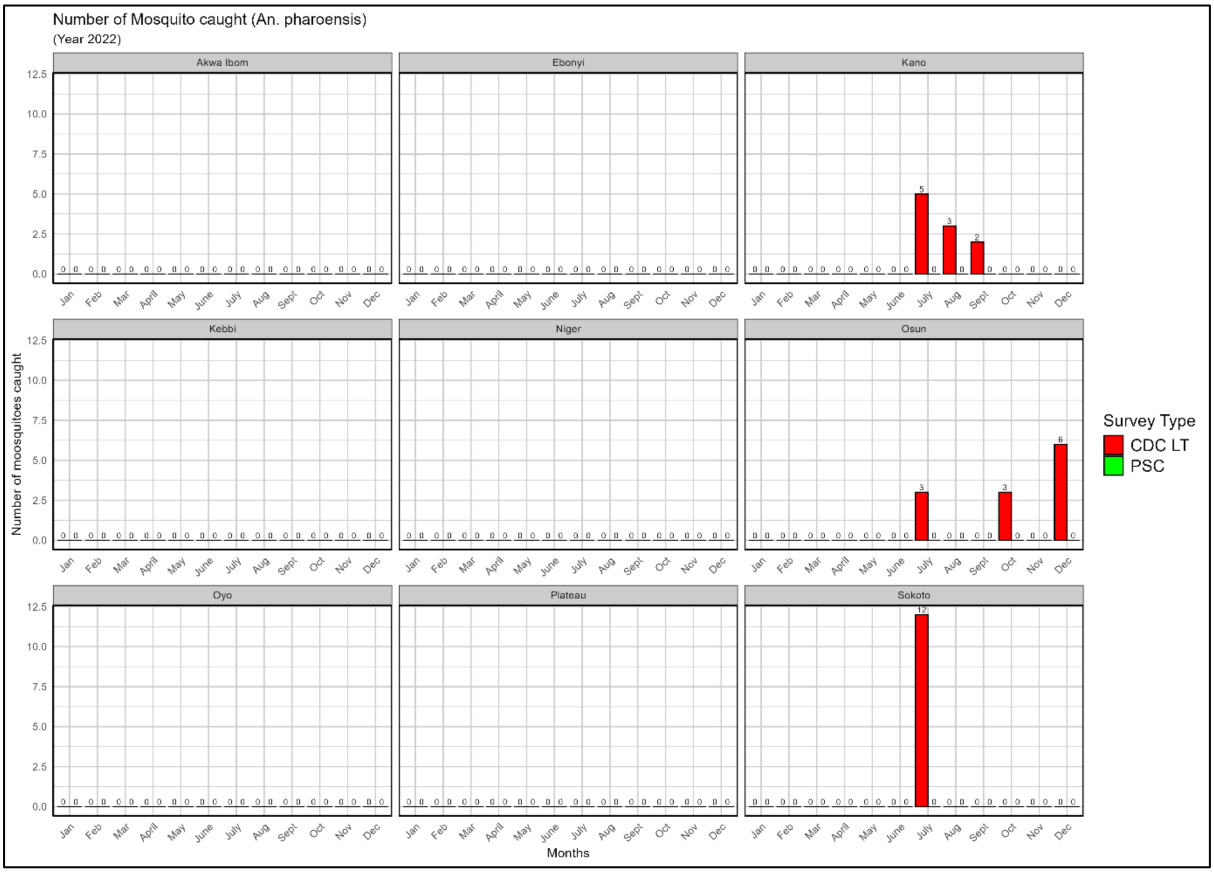

Figure 15: Monthly catches of *An. pharoensis* for CDC LT and PSC methods of collection for Year 2022

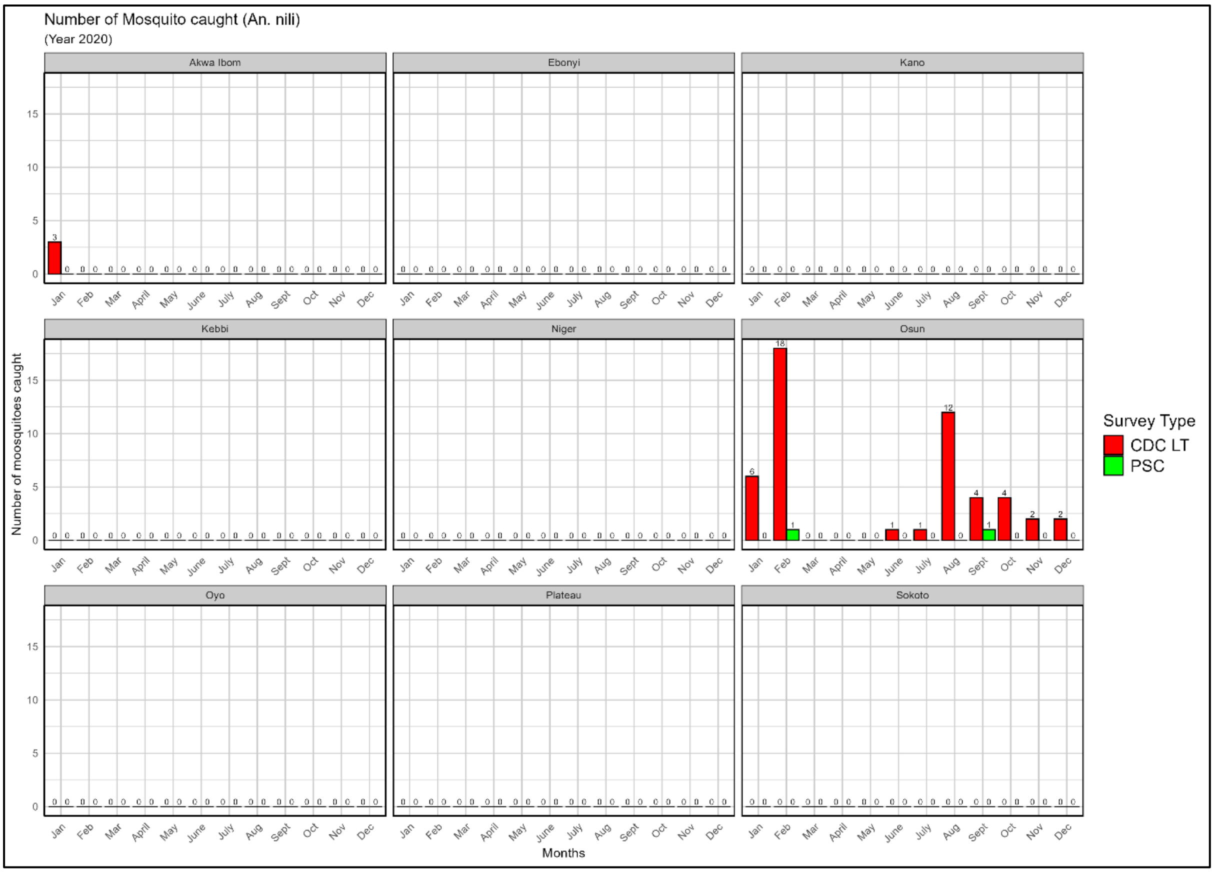

Figure 16: Monthly catches of *An. nili* for CDC LT and PSC methods of collection for Year 2020

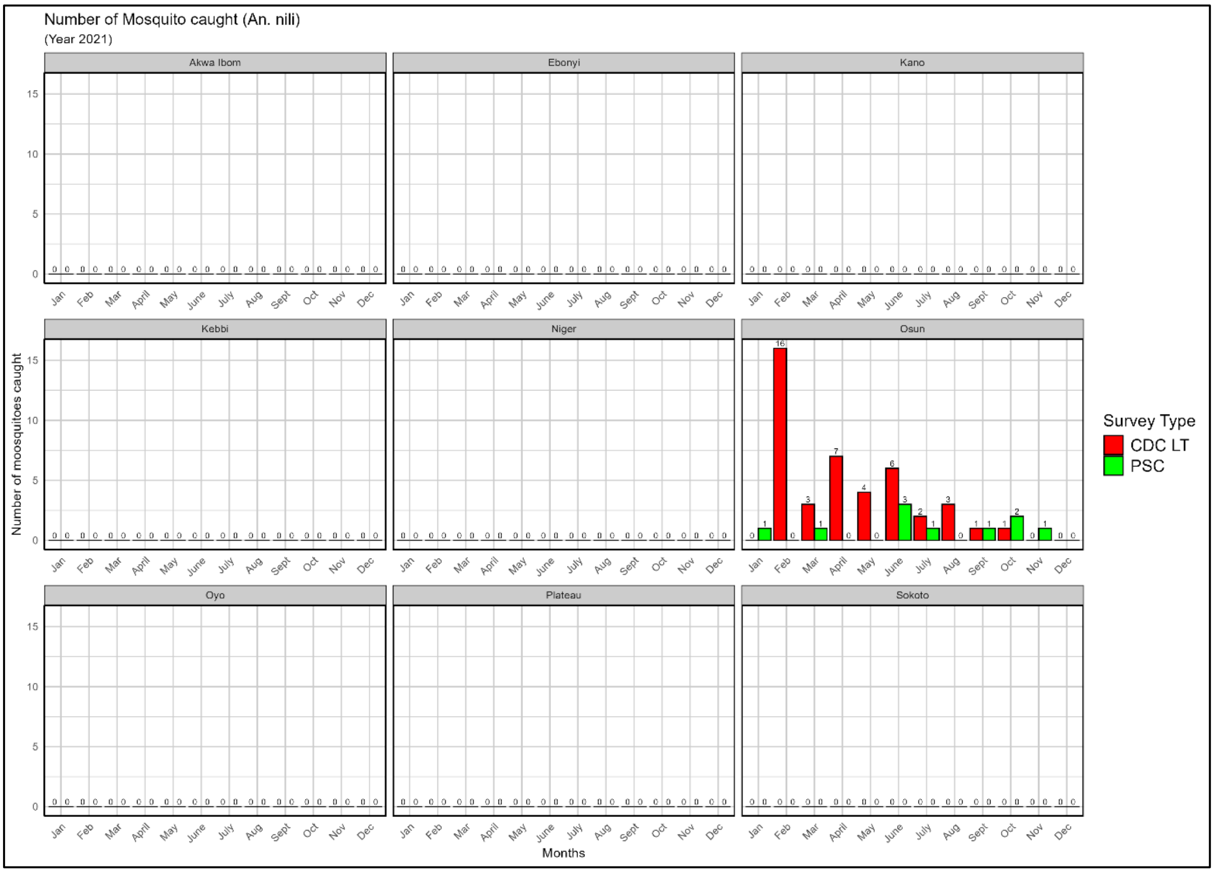

Figure 17: Monthly catches of *An. nili* for CDC LT and PSC methods of collection for Year 2021

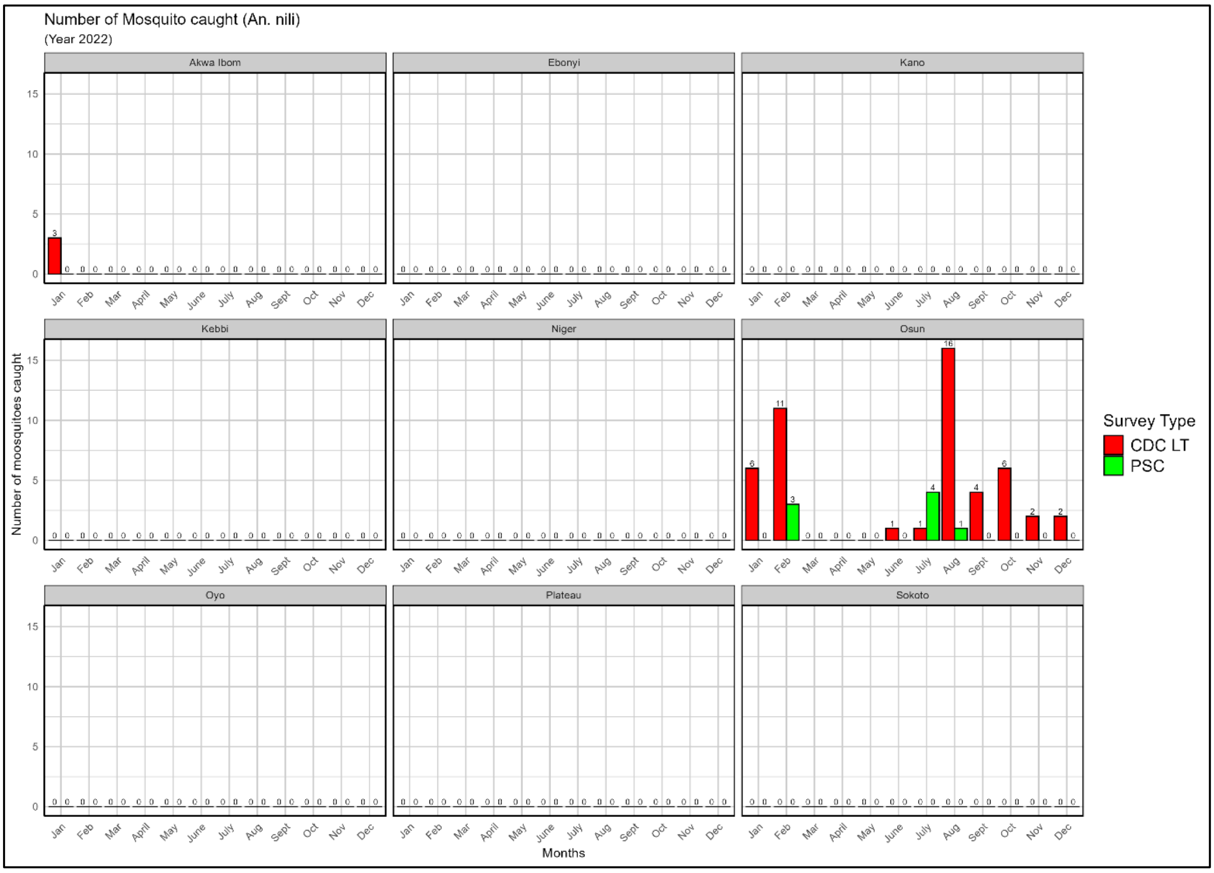

Figure 18: Monthly catches of *An. nili* for CDC LT and PSC methods of collection for Year 2022

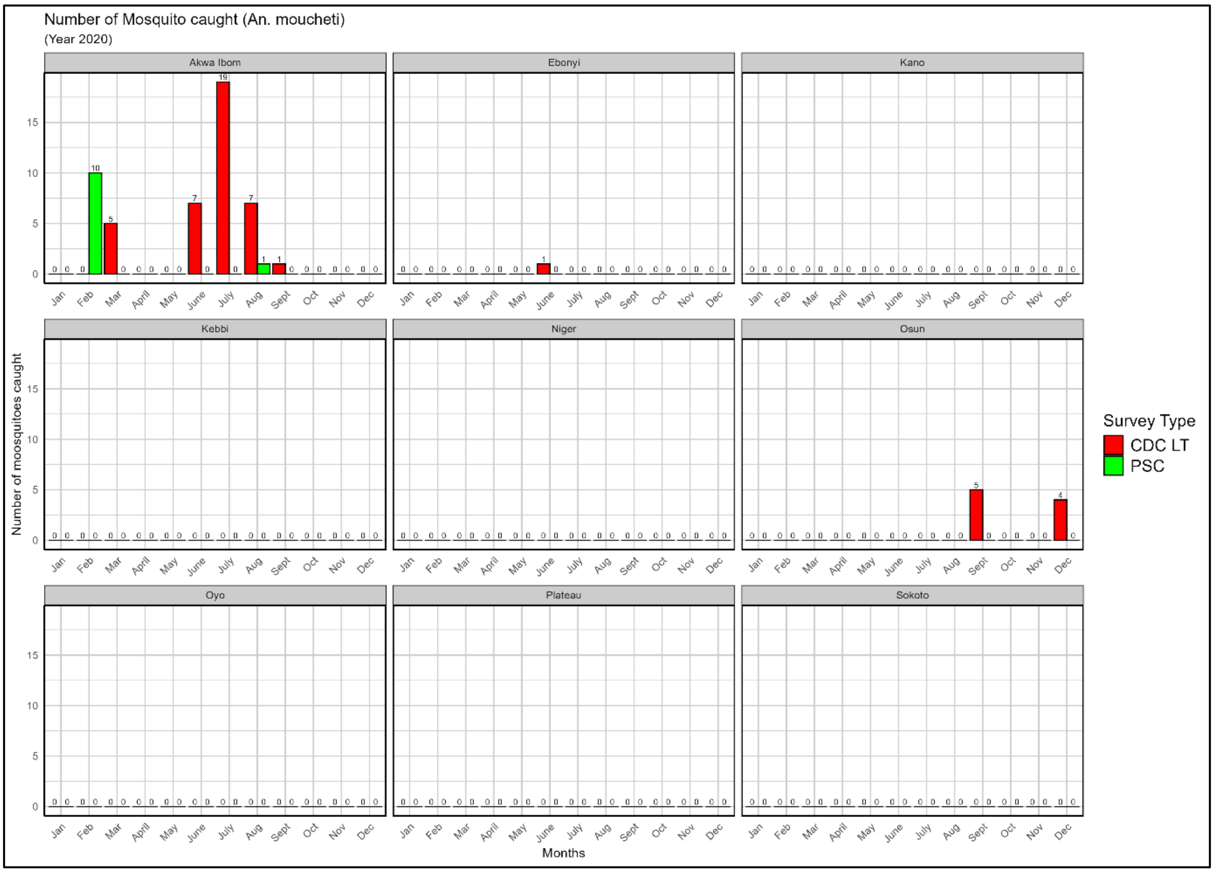

Figure 19: Monthly catches of *An. moucheti* for CDC LT and PSC methods of collection for Year 2020

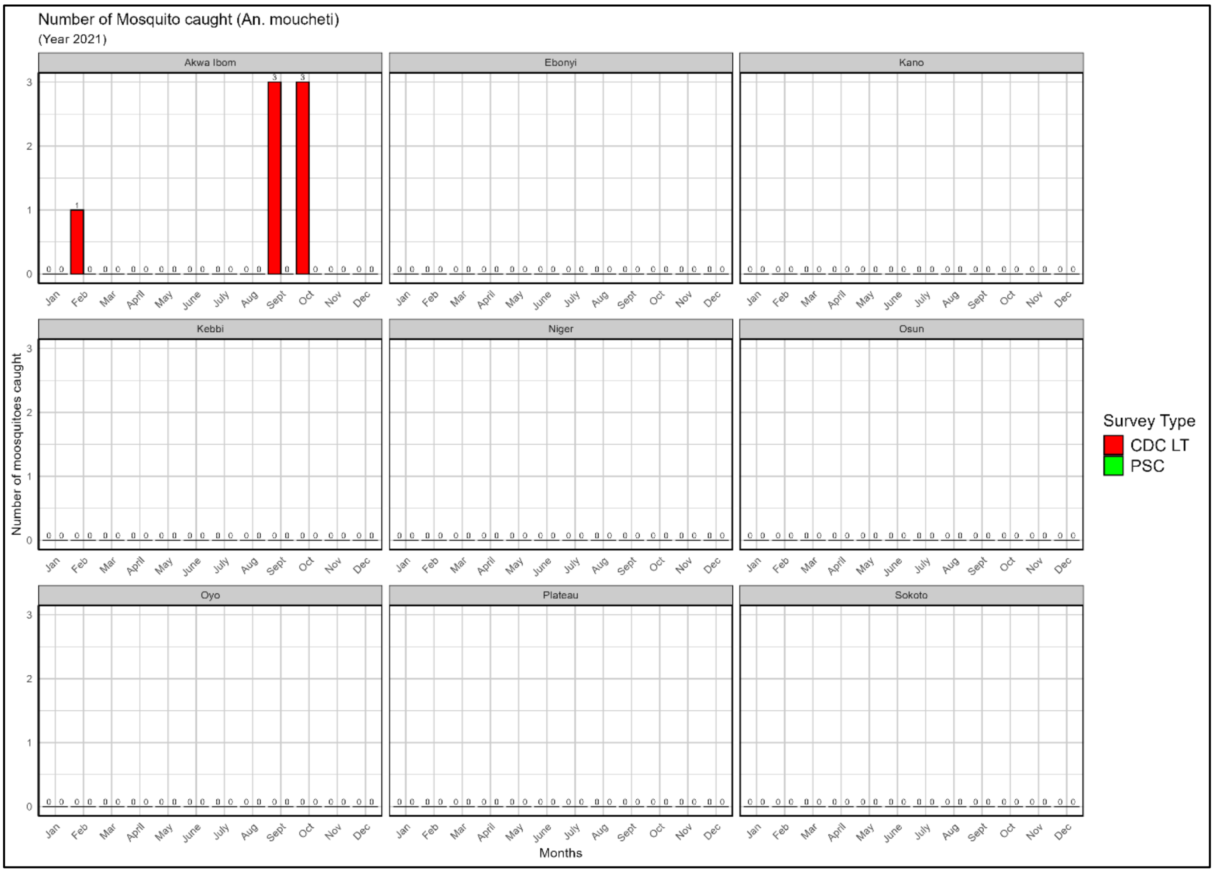

Figure 20: Monthly catches of *An. moucheti* for CDC LT and PSC methods of collection for Year 2021

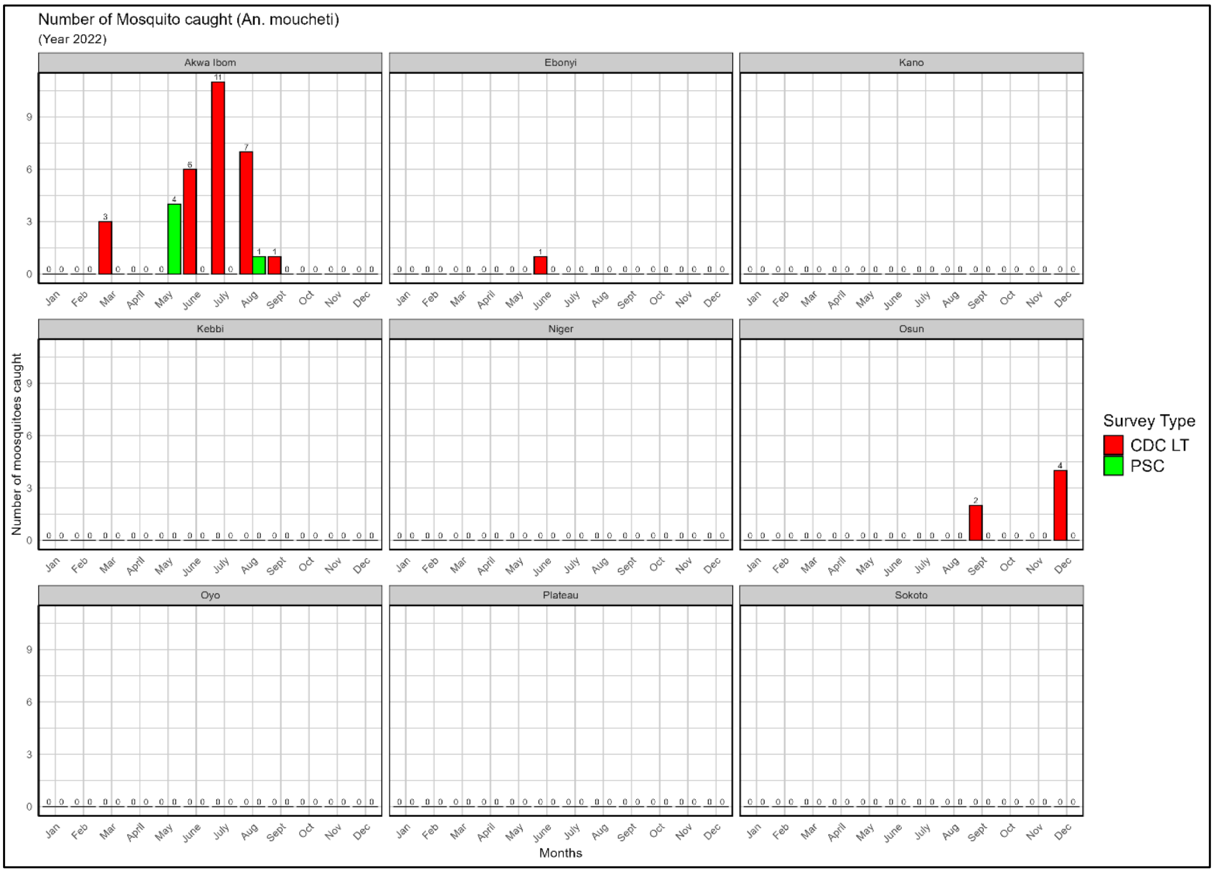

Figure 21: Monthly catches of *An. moucheti* for CDC LT and PSC methods of collection for Year 2022

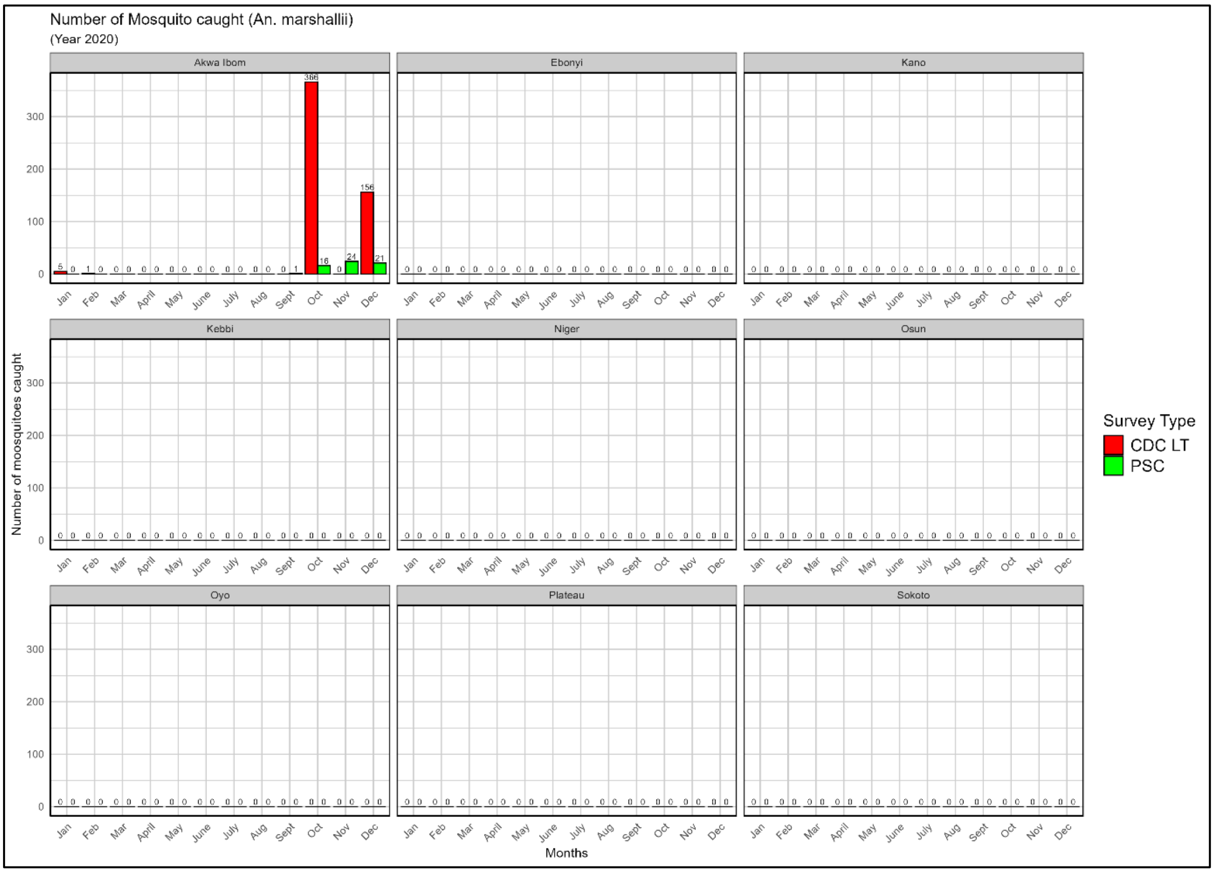

Figure 22: Monthly catches of *An. marshalliii* for CDC LT and PSC methods of collection for Year 2020

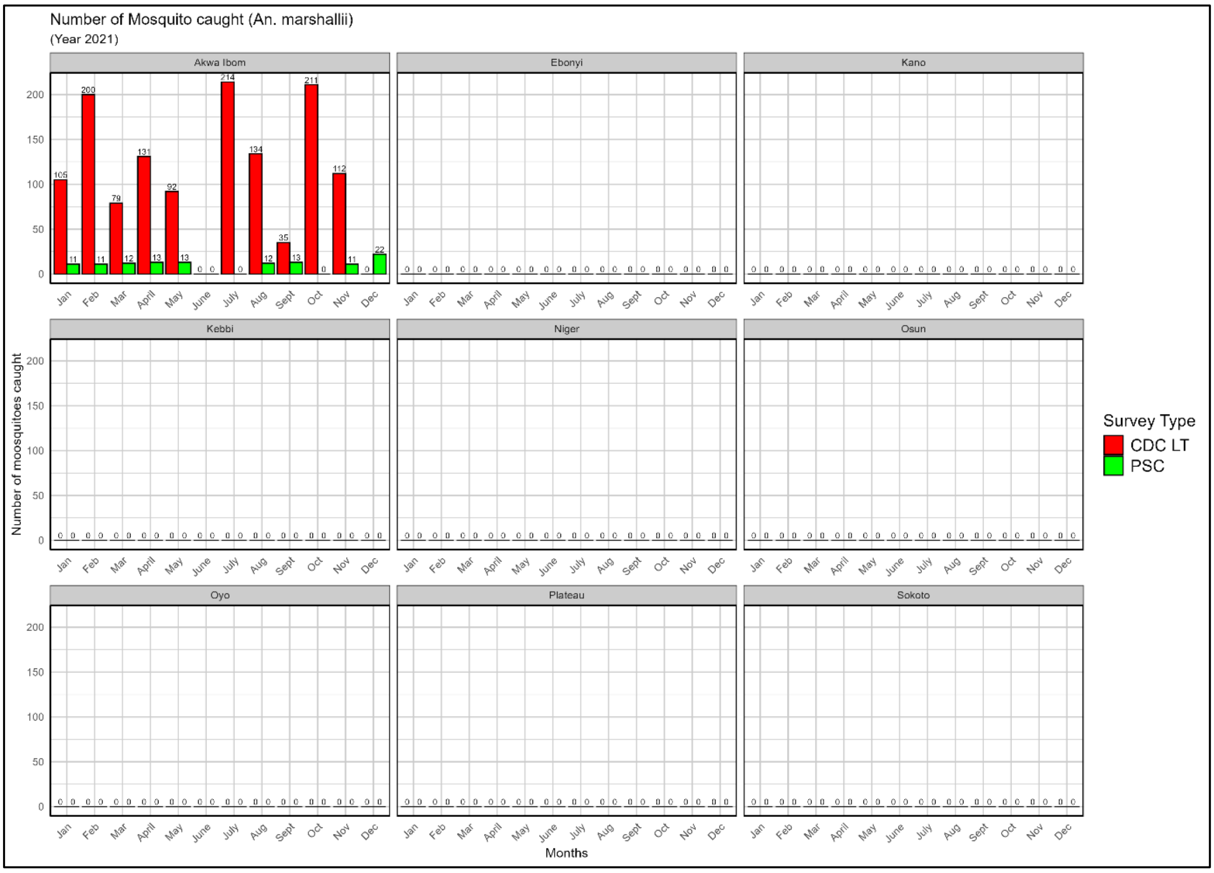

Figure 23: Monthly catches of *An. marshalliii* for CDC LT and PSC methods of collection for Year 2021

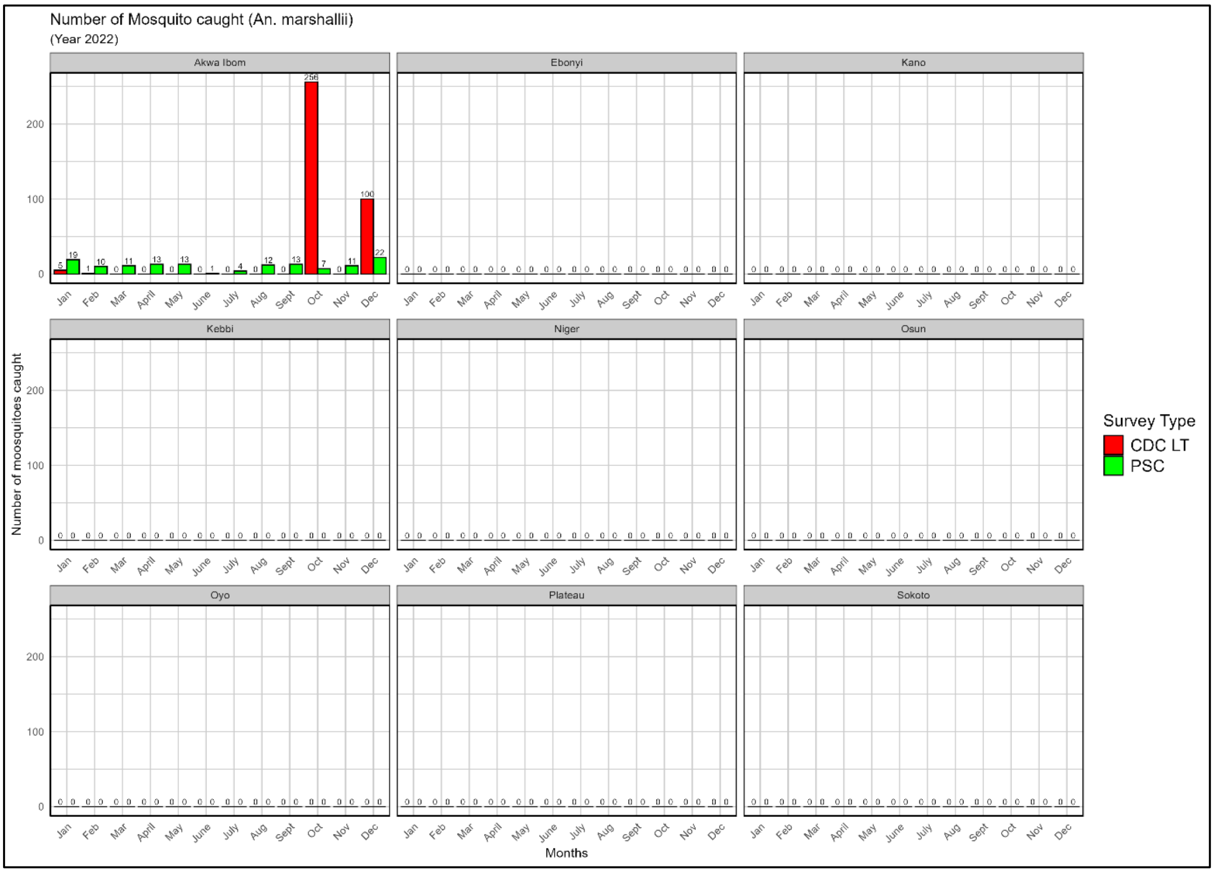

Figure 24: Monthly catches of *An. marshalliii* for CDC LT and PSC methods of collection for Year 2022

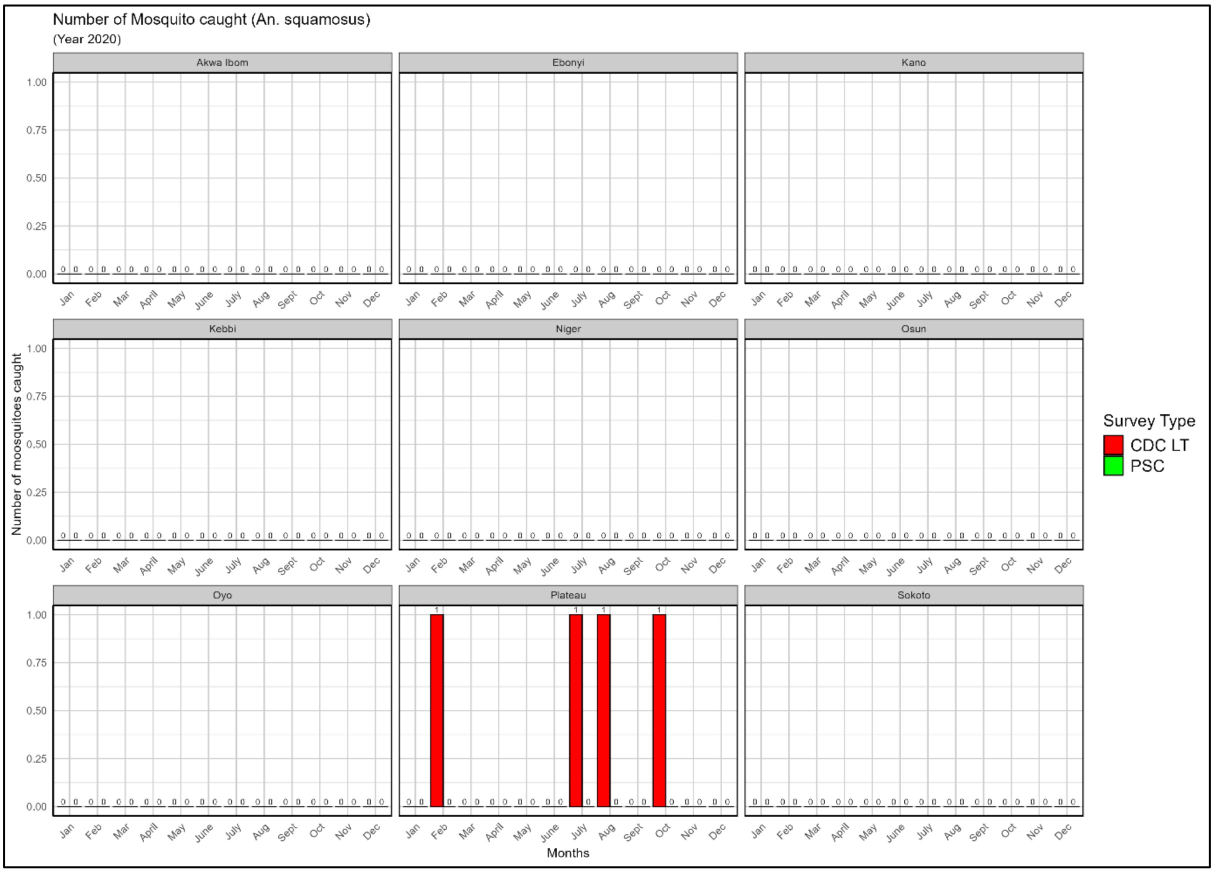

Figure 25: Monthly catches of *An. sqamosus* for CDC LT and PSC methods of collection for Year 2020

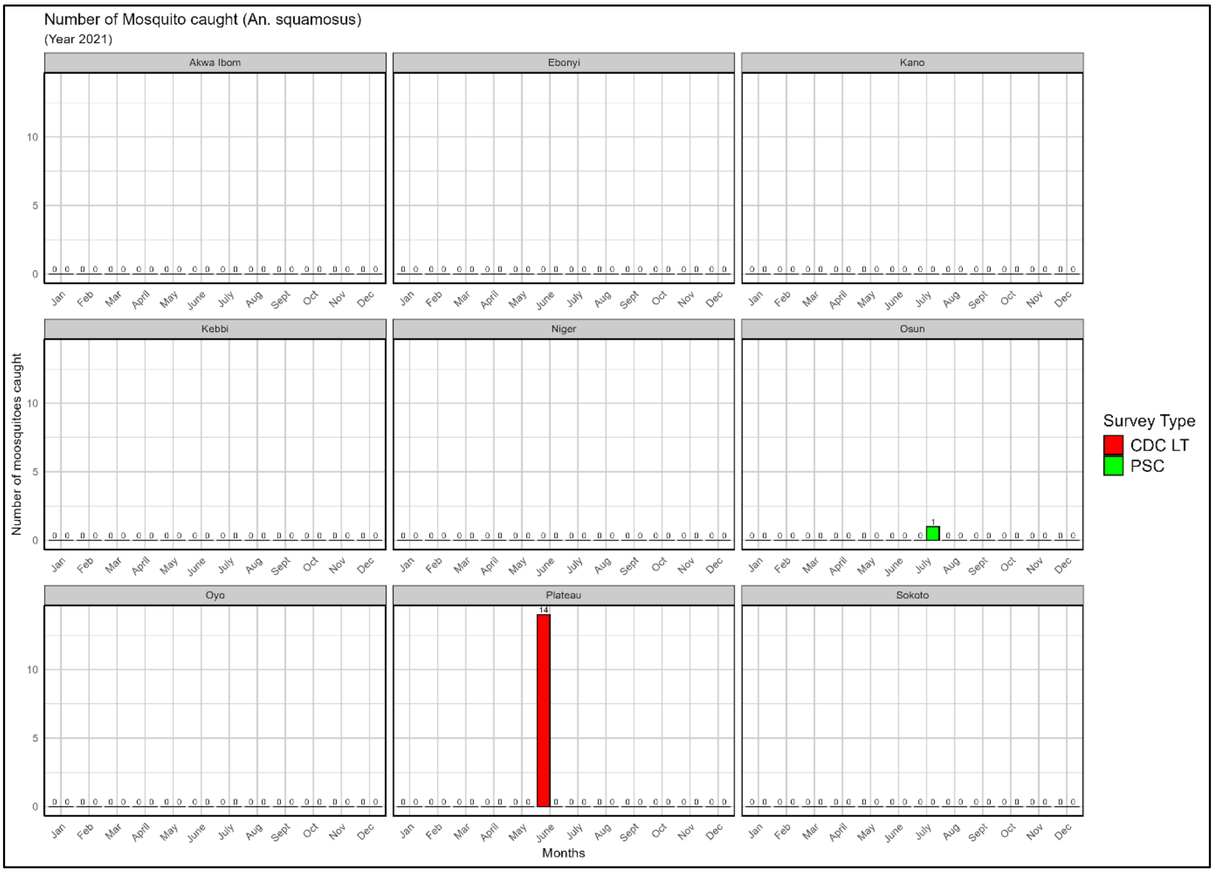

Figure 26: Monthly catches of *An. sqamosus* for CDC LT and PSC methods of collection for Year 2021

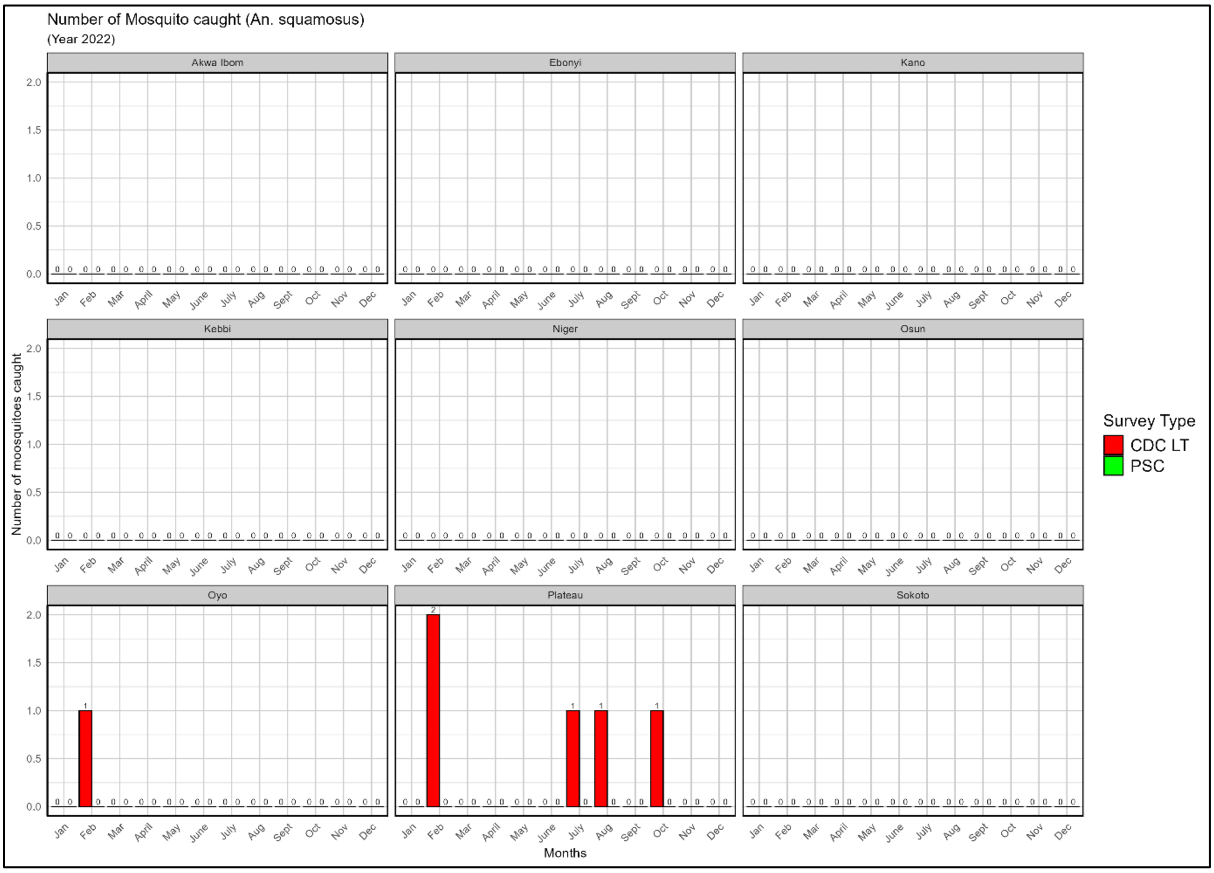

Figure 27: Monthly catches of *An. sqamosus* for CDC LT and PSC methods of collection for Year 2022

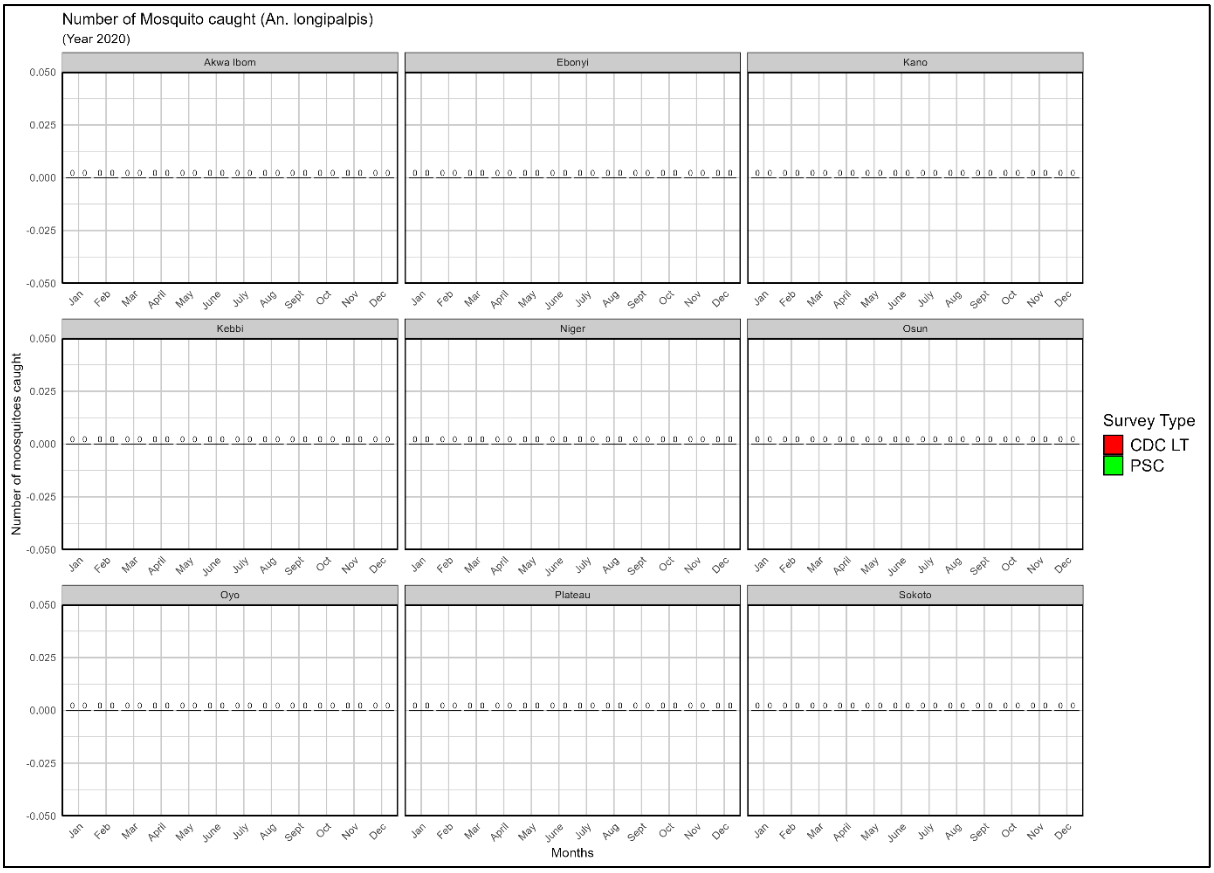

Figure 28: Monthly catches of *An.longipalpis* for CDC LT and PSC methods of collection for Year 2020

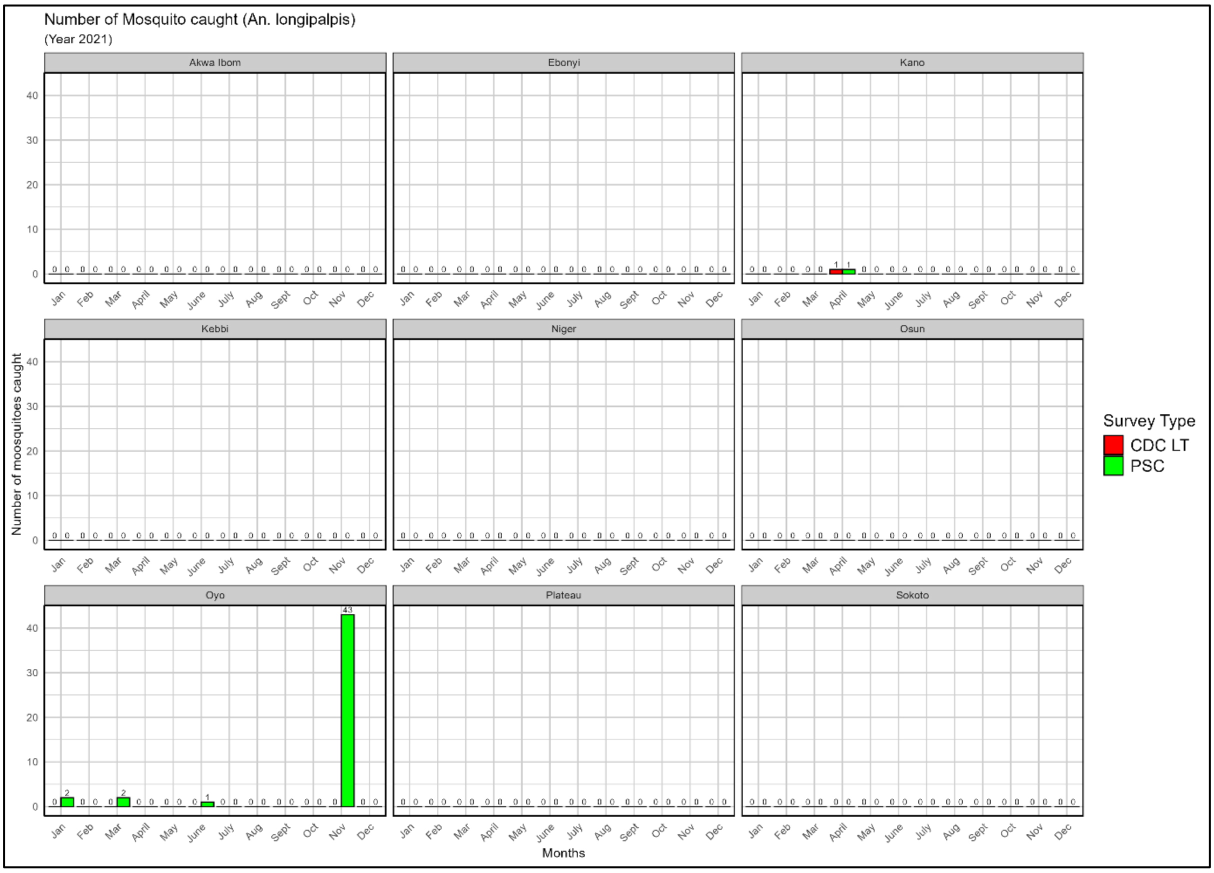

Figure 29: Monthly catches of *An.longipalpis* for CDC LT and PSC methods of collection for Year 2021

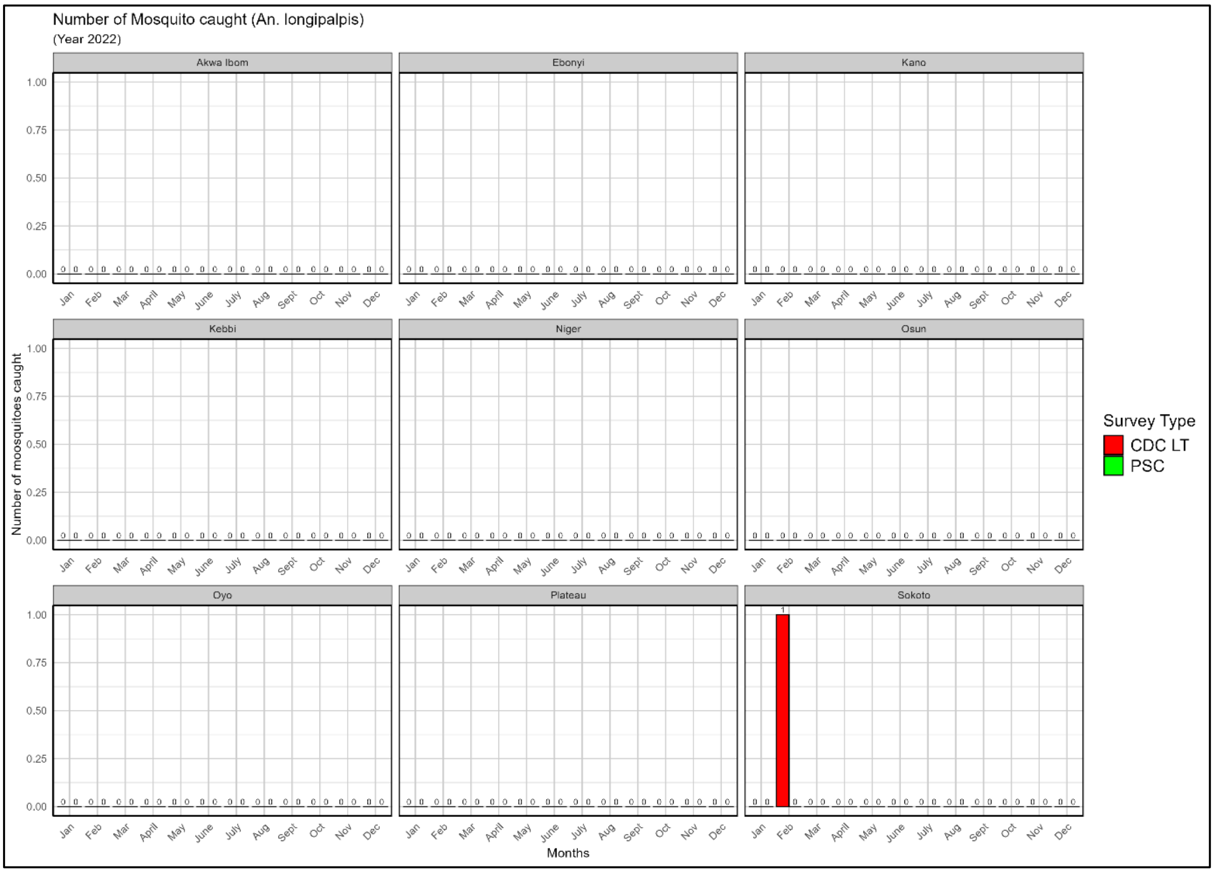

Figure 30: Monthly catches of *An.longipalpis* for CDC LT and PSC methods of collection for Year 2022
